# Double Optimization of Rivastigmine-Loaded Nanostructured Lipid Carriers (NLC) for Nose-to-Brain Delivery Using the Quality by Design (QbD) Approach: Formulation Variables and Instrumental Parameters

**DOI:** 10.3390/pharmaceutics12070599

**Published:** 2020-06-28

**Authors:** Sara Cunha, Cláudia Pina Costa, Joana A. Loureiro, Jorge Alves, Andreia F. Peixoto, Ben Forbes, José Manuel Sousa Lobo, Ana Catarina Silva

**Affiliations:** 1 UCIBIO/REQUIMTE, MEDTECH Laboratory of Pharmaceutical Technology, Department of Drug Sciences, Faculty of Pharmacy, University of Porto, 4050-313 Porto, Portugal; up201510339@ff.up.pt (S.C.); claudiasofiapcosta@gmail.com (C.P.C.); slobo@ff.up.pt (J.M.S.L.); 2 LEPABE, Department of Chemical Engineering, Faculty of Engineering, University of Porto, 4200-465 Porto, Portugal; up200505519@fe.up.pt; 3 Thermo Unicam, 4470-108 Porto, Portugal; jorge.alves@thermounicam.pt; 4 LAQV/REQUIMTE, Department of Chemistry and Biochemistry, Faculty of Sciences, University of Porto, 4169-007 Porto, Portugal; andreia.peixoto@fc.up.pt; 5 Institute of Pharmaceutical Science, Faculty of Life Sciences and Medicine, King’s College London, London SE1 9NH, UK; ben.forbes@kcl.ac.uk; 6 UFP Energy, Environment and Health Research Unit (FP ENAS), Fernando Pessoa University, 4249-004 Porto, Portugal

**Keywords:** nanostructured lipid carriers (NLC), formulation optimization, rivastigmine, quality by design (QbD), nasal route, nose-to-brain

## Abstract

Rivastigmine is a drug commonly used in the management of Alzheimer’s disease that shows bioavailability problems. To overcome this, the use of nanosystems, such as nanostructured lipid carriers (NLC), administered through alternative routes seems promising. In this work, we performed a double optimization of a rivastigmine-loaded NLC formulation for direct drug delivery from the nose to the brain using the quality by design (QbD) approach, whereby the quality target product profile (QTPP) was the requisite for nose to brain delivery. The experiments started with the optimization of the formulation variables (or critical material attributes—CMAs) using a central composite design. The rivastigmine-loaded NLC formulations with the best critical quality attributes (CQAs) of particle size, polydispersity index (PDI), zeta potential (ZP), and encapsulation efficiency (EE) were selected for the second optimization, which was related to the production methods (ultrasound technique and high-pressure homogenization). The most suitable instrumental parameters for the production of NLC were analyzed through a Box–Behnken design, with the same CQAs being evaluated for the first optimization. For the second part of the optimization studies, were selected two rivastigmine-loaded NLC formulations: one produced by ultrasound technique and the other by the high-pressure homogenization (HPH) method. Afterwards, the pH and osmolarity of these formulations were adjusted to the physiological nasal mucosa values and in vitro drug release studies were performed. The results of the first part of the optimization showed that the most adequate ratios of lipids and surfactants were 7.49:1.94 and 4.5:0.5 (%, *w*/*w*), respectively. From the second part of the optimization, the results for the particle size, PDI, ZP, and EE of the rivastigmine-loaded NLC formulations produced by ultrasound technique and HPH method were, respectively, 114.0 ± 1.9 nm and 109.0 ± 0.9 nm; 0.221 ± 0.003 and 0.196 ± 0.007; −30.6 ± 0.3 mV and −30.5 ± 0.3 mV; 97.0 ± 0.5% and 97.2 ± 0.3%. Herein, the HPH was selected as the most suitable production method, although the ultrasound technique has also shown effectiveness. In addition, no significant changes in CQAs were observed after 90 days of storage of the formulations at different temperatures. In vitro studies showed that the release of rivastigmine followed a non-Fickian mechanism, with an initial fast drug release followed by a prolonged release over 48 h. This study has optimized a rivastigmine-loaded NLC formulation produced by the HPH method for nose-to-brain delivery of rivastigmine. The next step is for in vitro and in vivo experiments to demonstrate preclinical efficacy and safety. QbD was demonstrated to be a useful approach for the optimization of NLC formulations for which specific physicochemical requisites can be identified.

## 1. Introduction

Alzheimer’s disease is an irreversible neurodegenerative disorder characterized by neuronal deterioration that leads to the loss of cognitive functions [1,2,3]. Genetic, environmental, and aging factors; the presence of neurofibrillary tangles; and senile plaques in the brain caused by the agglomeration of poorly folded proteins have been highlighted as the main factors involved in Alzheimer’s pathogenesis [1,2,4]. Drugs used in clinical practice can attenuate the disease symptoms, inhibiting acetylcholinesterase activity and avoiding acetylcholine hydrolysis in the synaptic cleft [5,6]. Examples of these drugs include galantamine, donepezil, and rivastigmine [1,7]. Among these, rivastigmine hydrogen tartrate, chemically known as (S)-N-ethyl-N-methyl-3-[(1-dimethylamino) ethyl]-phenyl carbamate hydrogen tartrate, is the most used as a reversible non-competitive dual inhibitor of acetylcholinesterase and butyrylcholinesterase, improving central cholinergic function through the increase of acetylcholine levels [7,8,9]. Nonetheless, it was reported that rivastigmine hydrogen tartrate undergoes an extensive first-pass effect in the liver, which decreases bioavailability [10,11]. This molecule also has a short half-life and a hydrophilic nature, which makes it difficult for it to pass through the blood brain barrier (BBB) and cerebrospinal fluid (CSF) [5,9,12]. In addition, the tight junctions between the BBB capillary endothelial cells restrict the passage, absorption, and permeation of drugs to the brain [13,14]. Therefore, high drug concentration and frequent dose administration are required to reach therapeutic levels, causing unpleasant cholinergic side effects, such as nausea, dyspepsia, bradycardia, and hallucinations [8,10,11].

More effective ways of delivering rivastigmine to the brain are required, such as the use of nanosystems administered through alternative administration routes [11,13,15,16,17,18]. Herein, the intranasal route has been considered for delivering drugs from the nose directly to the brain, avoiding the need to overcome the BBB [13,19,20]. The nasal cavity directly contacts the central nervous system (CNS) through the olfactory and trigeminal nerves that connect to the brain and the CSF, allowing direct drug transport [13,20,21,22]. The nasal route offers other advantages to improve drug delivery, including the avoidance of the first-pass effect and gastric degradation, high drug absorption, and reduction of adverse effects. However, this route shows some limitations, such as fast drug elimination by the mucociliary clearance mechanism, among others [11,19,23,24,25]. Notwithstanding, the composition of the nasal formulation is crucial to obtain high therapeutic efficiency, being influenced by excipients, the physical state of the dosage form, and the applied volume [23,24].

Regarding nanosystems, several studies have showed that they promote nasal delivery, providing sustained drug release while avoiding molecules degradation due to the protective shell [11,23]. In this area, lipid nanoparticles (solid lipid nanoparticles—SLN; nanostructured lipid carriers—NLC) have shown high potential as carriers for nose-to-brain drug delivery [13,17,18,26,27,28]. SLN and NLC seem more advantageous than other nanosystems for brain delivery, as they are made of physiological lipids and are generally recognized as safe (GRAS) excipients that are biocompatible and biodegradable [13,26,29,30]. Furthermore, they provide drug protection against enzymatic degradation and increase the residence time in the nasal cavity, improving drug bioavailability [14,17,25]. Other advantages include the ease of production on a large scale without the use of organic solvents, the high encapsulation efficiency for lipophilic molecules, and having a controlled release profile [13,15,22,30,31,32]. Besides, it is possible to produce SLN and NLC with diameters below 200 nm and a polydispersity index (PDI) of around 0.3, which are recommended for nose-to-brain delivery [13,31,33,34].

Although the clinical use of nanosystems has been intensively studied, some specific regulatory requirements are lacking [35]. In this sense, the use of the quality by design (QbD) approach to optimize lipid nanoparticles is essential to design formulations with low risk of failure and to achieve the desired clinical attributes. Thereby, carrying out preliminary studies to ensure the quality of the final product is required to achieve high efficiency, stability, and reproducibility. Some of these studies have explored the definition of the desired administration route and drug release profile, followed by the evaluation of the formulation properties and the control of the variables of the production method [36,37].

The Food and Drug Administration (FDA) and European Medicines Agency (EMA) authorities have encouraged the use of the QbD approach as a continuous process that should be applied to the development of a new pharmaceutical product, defining the quality target product profile (QTPP) to obtain a final product with high quality, safety, and efficiency [38,39]. QbD starts with the selection of the critical process parameters (CPPs) and critical material attributes (CMAs) that interfere with the critical quality attributes (CQAs), which are based on risk management [38]. For the implementation and continuous improvement of the QbD approach, several quality tools described in the International Council for Harmonisation of Technical Requirements for Pharmaceuticals for Human Use (ICH) Q8, Q9, and Q10 guidelines are used, namely the Ishikawa diagram, Pareto chart, response surface methodology, and design of experiment (DoE) tools [40,41]. These tools are fundamental in the optimization of formulations, reducing the number of required experiments and consequently saving time and costs [11,32].

The aim of this work was to use the QbD approach to optimize a rivastigmine-loaded NLC formulation for nose-to-brain delivery with the predefined QTPP for the particle size (<200 nm), PDI (<0.3), zeta potential (ZP) (close to ±30 mV), and encapsulation efficiency (EE) (>80%) [31,33,34,42]. To carry out a complete and accurate optimization of the formulation, the study was divided into two parts. First, the most suitable CMAs, which corresponded to the concentrations of the different formulation components (lipids and surfactants), were defined. Afterwards, the CPPs were selected, which corresponded to the production method (high-pressure homogenization—HPH; or ultrasound technique) to produce rivastigmine-loaded NLC formulations with the desired QTPP. A central composite design was used to optimize the CMAs to achieve high quality predictions for various factors at extreme levels [32,43,44], and a Box–Behnken design was used to optimize the CPPs, analyzing the effects of three variables and requiring less experiments [45,46,47]. Finally, the pH and osmolarity of the optimized NLC formulations were adjusted to the physiological values and in vitro release studies were performed. Rivastigmine quantification was assessed by a high-performance liquid chromatography (HPLC) method validated according to the European Pharmacopeia (Ph. Eur.) and ICH guidelines [48,49]. In addition, the long-term stability of the optimized rivastigmine-loaded NLC formulations was assessed by measuring the particle size, PDI, ZP, and EE values over 90 days of storage at 20.0 ± 0.5 °C and 4.0 ± 0.5 °C.

## 2. Materials

Rivastigmine base (liquid) of 99.9% purity was kindly provided by Novartis (Basel, Switzerland). Precirol^®^ ATO 5 (glyceryl distearate/glyceryl palmitostearate) was donated from Gattefossé (Lyon, France) and Phospholipon^®^ 90G (phosphatidylcholine, hydrogenated) was a gift from Lipoid (Ludwigshafen am Rhein, Germany), alpha-tocopherol acetate (vitamin E), polysorbate 80 (Tween^®^ 80) and benzalkonium chloride were purchased from Acef (Piacenza, Italy) and Acofarma (Barcelona, Spain), respectively. The water used in all experiments was purified, obtained from a Milli^®^Q Plus, Millipore^®^ (Darmstadt, Germany). For the mobile phase, acetonitrile ≥99.9% purity was purchased from Fisher Chemical-Thermo Fisher Scientific (Loughborough, UK); disodium phosphate was purchased from Sigma Aldrich (Lisbon, Portugal); monosodium phosphate from Merck (Darmstadt, Germany); and sodium chloride, potassium chloride and calcium chloride were purchased from Acofarma (Barcelona, Spain).

## 3. Methods

### 3.1. Screening of Drug and Excipients

Prior to NLC production, it is mandatory to study the compatibility between solid and liquid lipids and between lipids and the drug to obtain a final formulation with high encapsulation efficiency and long-term stability [50,51]. The components of the NLC formulation were selected from previous work developed by our group [52,53]. Precirol^®^ ATO5 was used as the solid lipid, due to its appropriate melting point (56 °C) and ability to form the imperfect lipid matrix of the NLC when mixed with a liquid lipid, which provides a high drug loading capacity [54,55]. Vitamin E was selected as the liquid lipid due to its antioxidant activity, which can delay the neural damage caused by the oxidative stress of Alzheimer’s disease, improving the neuroprotective effect of the NLC formulation. In addition, vitamin E decreases the risk of lipid oxidation resulting from the preparation of NLC, increasing the chemical stability of the encapsulated drug; easily solubilizes lipophilic molecules; and has high compatibility with lipids and surfactants [56,57,58].

The compatibility of different amounts of vitamin E with the solid lipid (Precirol^®^ ATO5) was evaluated in ratios ranging from 50:50 up to 90:10 (solid lipid: liquid lipid, % *w*/*w*). For the experiments, the lipid mixture was heated up to 100 °C under stirring at 200 rpm for 1 h and cooled down to room temperature (25 ± 0.5 °C). The solidified mixture was then analyzed by passing through a filter paper, where the absence of oil stains indicated the existence of miscibility between the lipids. Afterwards, the best proportion of solid and lipid liquids was selected [59].

To study the compatibility between the drug and lipids, different amounts of the drug were added to the lipid mixture previously selected, using as reference the concentration of a commercial drug solution (2%, *w*/*w*). For the tests, increasing amounts of drug (0.1%, 0.2%, 0.5%, 1%, and 2%) were added to the lipids mixture and heated 10 °C above the melting point of the solid lipid (70 ± 0.5 °C) under stirring at 500 rpm for 1 h. After solidification by cooling to room temperature, the mixture was placed on a filter paper, where the absence of oil droplets indicated the existence of drug solubility in the lipid mixture [59].

SLN and NLC formulations should include two surfactants that promote steric and electrostatic stabilization, avoiding nanoparticle aggregation and ensuring long-term stability. Surfactants should be selected according to their charge, molecular weight, and adequacy for the desired route of administration for the formulation [22,50,60,61]. Smaller particle sizes have been observed when a higher surfactant/lipid ratio was used [31,62,63]. Accordingly, polysorbate 80 (Tween^®^ 80), a non-ionic surfactant containing a polyoxyethylene chain tetrahydrofuran ring that provides steric stabilization and a hydrophobic tail that prevents particle aggregation, was selected based on previous works that showed its compatibility with the lipids used [46,52,53,59,60,64]. The co-surfactant (Phospholipon^®^ 90G) was selected based on its emulsification capacity for the selected lipid mixture, its non-irritating effect on the nasal mucosa, and its ability to minimize the polymorphic state transitions of lipids. Phospholipon^®^ 90G is a (phosphatidylcholine hydrogenated) biological membrane lipid and an amphoteric surfactant that has a synergic effect with Tween^®^ 80, originating NLCs with smaller particle sizes and high stability [64,65]. Different proportions of surfactant and co-surfactant were used to prepare NLC formulations (Table 1) and the best ratio was selected after analysis of the results of particle size, PDI, ZP, and EE tests (Section 3.5) [32].

Benzalkonium chloride, a quaternary ammonium compound, was used as preservative (0.02%) to prevent microbial proliferation of the NLC formulations due to the high water content. This compound is commonly used in nasal formulations as it exhibits low or no toxicity to the nasal cilia when used in concentrations between 0.01 and 0.02% [66].

Several research studies have described the use of similar components to prepare NLC formulations for nose-to-brain delivery. For instance, Khan et al. developed a hydrogel-containing temozolomide-loaded NLC for nose-to-brain delivery using vitamin E as the liquid lipid [67]. Madane et al. prepared a curcumin-loaded NLC for nose-to-brain delivery using Precirol^®^ ATO5, Tween^®^ 80, and lecithin (a phospholipid similar to Phospholipon^®^ 90G) [33]. Wavikar et al. used Tween^®^ 80 and lecithin to prepare a rivastigmine-loaded NLC for nose-to-brain delivery [68]. Precirol^®^ ATO5 and Tween^®^ 80 were used to prepare a NLC to improve the nose-to-brain transport of a glial cell-derived neurotrophic factor (GDNF) [69]. Tween 80^®^ was used as surfactant olanzapine-loaded NLC [70] and in an asenapine-loaded NLC to promote brain delivery through intranasal administration [71].

### 3.2. Preparation of Rivastigmine-Loaded NLC Formulations

Rivastigmine-loaded NLC formulations (Table 1) were prepared by HPH and ultrasound technique, which were previously employed by Silva et al. [18,53]. Briefly, the lipid phase was heated above the solid lipid melting point and added to the aqueous phase, which was previously heated at the same temperature. Afterwards, the mixture was emulsified under high-speed stirring with an Ultra-Turrax^®^ T25 (Janke and Kunkel GmbH, Staufen im Breisgau, Germany) at 13,400 rpm for 5 min. The oil-in-water (O/W) formed emulsion was sonicated by means of an VCX130 ultrasonic processor (Sonics, Wolfwil, Switzerland). The power output, with an amplitude of 75%, was applied for 15 min. The hot O/W nanoemulsion was transferred to glass vials and cooled to the room temperature (20 ± 0.5 °C) to form the NLC. A rivastigmine concentration of 0.12% (*w*/*w*) was added to the lipid phase before melting. Regarding the HPH, the procedure was similar, although the O/W emulsion was forced to pass through a piston gap homogenizer (Stansted High Pressure Homogenizer, Stansted Fluid Power Ltd., Harlow, UK) at 1750 bar and 80 ± 0.5 °C. The number of applied homogenization cycles ranged from 9 up to 18. The homogenizer was previously heated at 80 ± 0.5 °C with hot purified water and the temperature was kept constant to avoid lipid solidification.

### 3.3. Determination of Particle Size, Polydispersity Index (PDI), and Zeta Potential (ZP)

The NLC mean particle size (Z-Ave) and PDI were measured by dynamic light scattering (DLS) technique using a Malvern nanozetasizer (Malvern, UK). A refractive index of 1.46 and an absorption index of 0.001 were used for the lipids, while a refractive index of 1.330 was used for the solvent (water). In addition, the NLC electrical surface charge was assessed by laser doppler electrophoresis by means of ZP measurements using the same apparatus. The dispersions were diluted with ultrapure water and the ZP was calculated using the Helmholtz–Smoluchowski equation and run on the system software. The temperature was set at 25 ± 1 °C. Each sample was analyzed in five replicates (*n* = 5) and the results were reported as the mean ± standard deviation (SD).

To confirm the absence of microparticles, particle size was measured by laser diffraction using a Malvern Mastersizer 3000E (Malvern, UK). The used particle refractive index was 1.4, the absorption index was 0.001, and the water dispersant refractive was 1.33, with Mi’s theory being applied. The particle size was assessed by the values of the volume distribution (D50 and D90), indicating the percentage of particles with a diameter size equal or lower to the given values. The results were reported as the mean ± SD of five replicates (*n* = 5).

### 3.4. Rivastigmine Quantification

#### 3.4.1. Development and Validation of a High-Performance Liquid Chromatography (HPLC) Method

The wavelength of the maximum absorption (237 nm) of rivastigmine was selected by spectroscopy analysis using a Jasco V-650 UV-Vis spectrophotometer. To obtain drug peaks of suitable resolution, chromatographic conditions were tested, namely the composition of the mobile phase, flow rate, and injection volume.

#### 3.4.2. Chromatographic Conditions

An isocratic mobile phase consisting of a phosphate-buffered solution (pH 6.4) and acetonitrile (60:40, *v*/*v*) was vacuum filtered through a 0.45 µm membrane (Millipore^®^, Germany) and degassed by ultrasonication for 15 min. The flow rate of the mobile phase was 1.0 mL/min. Before sample injections, the system was cleaned with purified water for 60 min and was left to equilibrate with the mobile phase for 60 min. The oven was set at 25 ± 3 °C and UV detection was performed at 237 nm. For each analysis, sample volumes of 20 µL were injected in triplicate (*n* = 3).

#### 3.4.3. Preparation of Standard Solutions

A stock standard solution of rivastigmine (1200 µg/mL) was prepared by dissolving 0.12 g of the drug in acetonitrile using a 100 mL volumetric flask. Five working solutions (24, 48, 72, 120, 840 µg/mL) were prepared by diluting an adequate amount of stock standard solution with acetonitrile in a 25 mL volumetric flask. All analyses were performed in triplicate (*n* = 3). The method was validated according to the International Council for Harmonisation of Technical Requirements for Pharmaceuticals for Human Use (ICH) guidelines for linearity, precision, accuracy, specificity, and robustness [5,49]. The developed method was shown to be linear, precise, selective, and robust (Supplementary Data, Sections 1 and 2), and was used in the following studies.

#### 3.4.4. Assessment of Encapsulation Parameters

The effectiveness of lipid nanoparticles for drug incorporation can be assessed by calculating the encapsulation efficiency (EE) and loading capacity (LC). High EE and LC values suggest that lipid nanoparticles can encapsulate and delivery the desired therapeutic amount of drug, reducing adverse effects and frequency of administration [18].

EE and LC were determined indirectly by calculating the amount of free rivastigmine (non-encapsulated) in the aqueous phase of NLC dispersions according to the following equations [72]:

(1) $$\mathrm{EE}\;(\%)=\frac{\mathrm{Total}\;\mathrm{amount}\;\mathrm{of}\;\mathrm{rivastigmine}-\mathrm{amount}\;\mathrm{of}\;\mathrm{free}\;\mathrm{rivastigmine}}{\mathrm{Total}\;\mathrm{amount}\;\mathrm{of}\;\mathrm{rivastigmine}}\times 100$$

(2) $$\mathrm{LC}\;(\%)=\frac{\mathrm{Total}\;\mathrm{amount}\;\mathrm{of}\;\mathrm{rivastigmine}-\mathrm{Amount}\;\mathrm{of}\;\mathrm{free}\;\mathrm{rivastigmine}}{\mathrm{Total}\;\mathrm{amount}\;\mathrm{of}\;\mathrm{rivastigmine}-(\mathrm{Amount}\;\mathrm{of}\;\mathrm{free}\;\mathrm{rivastigmine}+\mathrm{Total}\;\mathrm{amount}\;\mathrm{of}\;\mathrm{lipid})}\times 100$$

Briefly, 1 mL of each sample was diluted with purified water and placed in an Amicon^®^ Ultracel-50K (Millipore Corporation, Ireland) centrifugal filter device and centrifuged at 3450 rpm for 1 h. Afterwards, the filtrate was collected, diluted in acetonitrile, and analyzed by HPLC. The tests were performed on the production day for 10 different batches of NLC formulations (*n* = 10) [72].

### 3.5. Design of Experiment (DoE) for the Optimization of Rivastigmine-Loaded NLC Formulation

A DoE was used to evaluate the effects of critical parameters related to the CMAs (i.e., formulation variables) and CPPs (i.e., instrumental parameters) on CQAs, namely for particle size, PDI, ZP, and EE.

**Figure 1:**
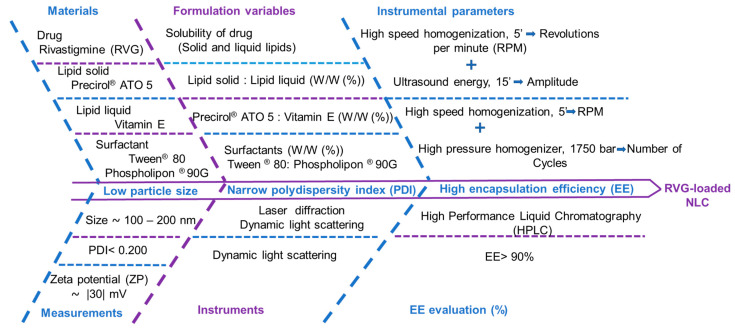
Ishikawa diagram showing the effects of critical material attributes (CMAs) and critical process parameters (CPPs) on the critical quality attributes (CQAs) of rivastigmine-loaded NLC formulation.

Figure 1 shows the Ishikawa diagram used as a visualization tool for the two parts of the optimization process.

#### 3.5.1. Part 1: Optimization of Formulation Variables by Central Composite Design (CCD)

In the first part of the optimization of the rivastigmine-loaded NLC formulation, we tested different solid lipid and liquid lipid (SL/LL) ratios, which were selected using the results of lipid-drug solubility tests and different ratios of surfactants.

The influence of the different ratios of SL/LL and surfactants on CQAs or dependent responses, namely particle size, PDI, ZP, and EE, were studied using a CCD with α rotatability of 1.4142, two-factors, and 3 levels. Table 1 shows the DoE used to test the rivastigmine-loaded NLC formulation variables.

Six DoE with 10 experimental runs were generated by the statistic software with two factors or independent variables corresponding to the CMAs, namely solid lipids (Precirol^®^ ATO 5, SL), vitamin E (LL), and surfactants (Tween^®^ 80 and Phospholipon^®^ 90G, Tw/Ph). Their effects on CQAs or dependent responses were studied at low (−1), medium (0), and high (+1) levels.

For the experiments, rivastigmine-loaded NLC formulations were produced by employing the ultrasound technique previous described by Silva et al. [72], involving high-speed homogenization at 13400 rpm and a sonication amplitude of 75%. The formulation with the most suitable values for CQAs, namely having a lower particle size, PDI of around 0.2–0.3, a ZP value close to 30mv, and an EE value > 90%, was selected for the next part of the optimization, which is related to the instrumental parameters [31,33,34,42]. It is important to note that ZP values close to │30│ mv are desired to ensure more stable NLC formulations, since the electrostatic repulsion between the nanoparticles prevents aggregation. However, negative ZP values reduce the residence time of the formulation in the nasal mucosa, since interactions between NLC and mucin, a negatively charged glycoprotein of the nasal cavity, may not occur [73,74]. To overcome this limitation, mucoadhesive polymers were added to the optimized rivastigmine-loaded NLC formulations to form in situ gels without negative charge. This strategy was used to develop NLC formulations for nose-to-brain delivery. For example, Rajput and Butani developed a NLC-based in situ gel for intranasal administration of resveratrol [75], while Abouhussein et al. developed a NLC-based in situ gel to improve the brain target of rivastigmine after intranasal administration [9].

#### 3.5.2. Part 2: Optimization of Instrumental Parameters by Box–Behnken Design (BBD)

The second part of the optimization aimed to study the effect of different CPPs in the CQAs or dependent responses that were evaluated in the first part of the optimization related to the selection of the most suitable concentrations of formulation components. The rivastigmine-loaded NLC formulation was produced by ultrasound technique and HPH, and the tested instrumental parameters were the emulsification speed (rpm), amplitude of sonication, and number of HPH cycles.

The emulsification time and speed are important parameters in obtaining small nanoparticles with a narrow PDI [11,46]. Thus, the emulsification time was set to 5 min and the effect of increasing the rpm on CQAs was evaluated. The time and amplitude of sonication are also important parameters in producing small NLCs. Generally, as the time and amplitude of sonication increase, particle size decreases [11,51]. However, it has been reported that a high amplitude of sonication increases the lipid nanoparticle size due to the formation of aggregates [11,46]. Therefore, the effect of increasing the amplitude of sonication on CQAs was evaluated.

Regarding HPH, the pressure was kept constant at 1750 bar, which allows the reduction of the particle size due to the generated cavitation forces. However, to obtain small and uniform nanoparticles, several homogenization cycles should be performed [61,76,77,78,79]. Thus, the effect of increasing the number of HPH cycles on CQAs was evaluated. The emulsification speed, sonication amplitude, and number of HPH cycles were the independent variables that were studied at low (−1), medium (0), and high levels (+1). The DoE showing the different combinations of the tested CPPs is presented in Table 2. For these studies, 9 experimental runs were performed for each of the 6 selected formulations to evaluate the effect of each independent variable on the particle size, PDI, ZP, and EE.

### 3.6. pH and Osmolarity

Regarding the requirements for nasal formulations, the pH (5.5–6.59) and osmolarity (280 mOsm/kg) were adjusted to the physiological values in the optimized rivastigmine-loaded NLC formulations [80].

The pH was measured at room temperature using a BASIS 20 calibrated digital pH meter (Crison Instruments, Spain) and the osmolarity was assessed using a Type 6 osmometer (Löser Messtechnik, Berlin-Spandau, Germany).

### 3.7. In Vitro Drug Release Studies

Drug release studies were carried out through dialysis bag diffusion technique over 48 h, as previous described by Silva et al. and Abouhussein et al. [9,81]. Simulated nasal electrolyte solution (SNES) and phosphate-buffered solution (pH 6.4) were used as release media. SNES was prepared by dissolving 12.9 mg of potassium chloride, 745 mg of sodium chloride, and 3.6 mg of calcium chloride in 1000 mL of ultrapure water. Phosphate-buffered solution pH 6.4 was prepared according to the European Pharmacopoeia (Ph. Eur.) [9,31,48]. The release profile of rivastigmine from the NLC was evaluated for the optimized formulations produced by HPH and ultrasound technique. Briefly, 2.5 mL of NLC with 1.2 mg/mL of rivastigmine were filled in a dialysis bag (cellulose membrane with molecular weight cut-offs of 300 kDa, 12–15 cm long, Spectra/Por^®^ Biotech, US), clamped, and immersed in a glass vial containing 250 mL of release medium at 37 ± 0.5 °C, then stirred at 50 rpm. At predetermined time intervals (0.5, 1, 2, 4, 6, 8, 10, 12, 24, 30, 36, and 48 h), 1.0 mL of sample was collected and the release medium was replaced with the same volume of fresh medium to guarantee sink conditions. Collected samples were passed through a syringe filter (0.21 µm) and diluted in 1 mL of acetonitrile, being the amount of rivastigmine measured by HPLC in a Thermo Scientific™ Dionex™ UltiMate™ instrument, with a detection wavelength of 237 nm using an analytical reverse-phase C_18_ column (100 mm × 4.6 mm, 5 µm) from Thermo Scientific Acclaim™ (Portugal). The results were reported as the mean ± SD of three replicates (*n* = 3). The cumulative rivastigmine released was calculated and expressed as a percentage of the theoretical maximum drug content.

#### Kinetic Mechanism of Drug Release

Drug release kinetics is a QTTP that should be considered in the development of a formulation, allowing the definition of in vivo–in vitro correlations [82,83]. In vitro drug release was analyzed by fitting the results to four mathematical kinetics models [72,83]: zero order (1), first order (2), Higuchi model (3), and Korsmeyer–Peppas (4) models. The correlation coefficient (*R*^2^) was determined to compare the precision of these models as it presents the highest *R*^2^ value, which was selected to describe the drug release kinetics. The value of the diffusion release exponent (*n*) obtained by the Korsmeyer–Peppas model was used to characterize the drug release mechanism [84,85]: *n* ≤ 0.43 means a Fickian release, where the drug diffusion is proportional to the concentration; *n* = 0.85 represents a non-Fickian release, i.e., a zero-order release, where the drug diffusion is independent from the concentration; 0.43 < *n* < 0.85 defines an anomalous transport route, which is a combination of non-Fickian release and Fickian release; *n* > 0.89 is a case II transport (relaxation-controlled release). The Microsoft Excel^®^ software was used to calculate the *R*^2^ and the model parameters of the following equations [86]:

(1) Zero order model: *M*_0_ − *M* = *kt*
(2) First order model: ln *m* = *kt*
(3) Higuchi equation: *M*_0_ − *M* = *kt*^1/2^
(4) Korsmeyer–Peppas model: log (*M*_0_ − *M*) = log *k* + *n* log *t*

*M* represents the amount of drug released at time *t*, *M*_0_ corresponds to the drug concentration at time 0, *k* is the rate constant, and *n* is the diffusion release exponent.

### 3.8. Statistical Analysis

Statistical analysis was performed using Statistica™ StatSoft software, version 13.5.0.17 (TIBCO^®^ Software Inc). Results were statistically analyzed by ANOVA and a 2-way interactions model, with a 95% confidence level being considered statistically significant when the value of *p* was less than 0.05. Pareto chart, contour, and 3-D response surface plots were used to select the best formulation variables and the ideal conditions of each instrumental parameter to achieve the desired CQA values.

### 3.9. Stability Studies

The long-term stability of the two optimized rivastigmine-loaded NLC formulations (i.e., the one with the best CQAs produced by the ultrasound technique and the one with the best CQAs produced by HPH method) was assessed according to ICH (Q1A) guidelines [87]. For the studies, formulations were stored at room temperature (20.0 ± 0.5 °C) and in the refrigerator (4.0 ± 0.5 °C), and the particle size (Z-Ave, D50, D90), PDI, ZP, and EE were evaluated after 90 days of storage. The results were presented as mean values of three replicates (*n* = 3) ± SD.

## 4. Results

### 4.1. Screening of Drug and Excipients

The selected lipids were compatible and miscible at the concentrations tested and a ratio of 70:30 (%, *w*/*w*) was shown to be the best proportions of Precirol^®^ ATO5 and vitamin E for the preparation of the NLC formulation, as previously developed in our group [52]. Several studies have shown that a high concentration of liquid lipids increases drug retention, as drug solubility in liquid lipids is usually higher than in solid lipids, which decreases the particle size due to the decreased viscosity and surface tension of the NLC [46,79,88].

The higher solubility of the drug in the lipid mixture was observed over individual lipids, due to the absence of oil droplets on the filter paper (Supplementary Data, Section 3.1, Figure S6). This can be explained by the imperfect lipid matrix formed in the mixture that allowed for higher amounts of drug molecules [46,79,88]. Accordingly, the selected drug concentration was 0.12%.

### 4.2. Suitability of the HPLC Method for Rivastigmine Quantification

The developed HPLC method was considered to be simple, linear, precise, selective, reproducible, and robust (Supplementary Data, Sections 1 and 2) for rivastigmine quantification. A good linearity (*R*^2^ = 0.999) was observed in the tested range, with RSD values lower than 1% and a recovery rate close to 100% being obtained. Furthermore, the values obtained for the detection and quantification limits were suitable for application of the method. The selectivity for rivastigmine quantification in the presence of other formulation components was also demonstrated and the results of encapsulation parameters showed that NLCs are effective for rivastigmine encapsulation.

### 4.3. Part 1: Optimization of Formulation Cariables by CCD

The rivastigmine-loaded NLC formulation variables were optimized using a CCD and the data were statistically analyzed using ANOVA, evaluating the importance of the selected factors on dependent responses and the suitability of the selected design for the CQAs through the value of R squared (*R*^2^). Different ANOVA models were tested (Supplementary Data, Section 3, Table S9) and the one that presented the closest *R*^2^ value to 1 (0.93563) for the CQAs was selected, namely the linear/quadratic main effects + 2-ways model [89,90].

#### 4.3.1. Effect of Lipids and Surfactants Ratio on Particle Size (Z-Ave, D50, and D90)

Table 3 shows that the predicted and observed values were very close, indicating the suitability of the design for the selected CQAs.

Predicted vs. observed plots showing the suitability of the model for the applied design, a Pareto chart of standardized effects studying the interaction effects of CMAs for each dependent response, and contour plots representing the interactions between CMAs and dependent responses were used for further analysis.

From the analysis of the Pareto chart (Supplementary Data, Section 3.1, Figure S4A), the surfactant ratio (Tw/Ph) and lipids ratio (SL/LL) were significant factors for particle size (Z-Ave) (*p* = 0.005), showing a negative effect, which means that particle size increased with increasing ratios of SL/LL and Tw/Ph. In contrast, for the dependent response D50 (Supplementary data, Figure S4B), Tw/Ph had a positive effect and the most statistically significant effect (*p* = 0.05) when a linear model was used, while SL/LL did not show a statistically significant effect. Regarding D90 (Supplementary data, Section 3.1, Figure S4C), it was observed that all independent variables had statistical significance (*p* = 0.05), showing positive effects on size when a linear model was applied, indicating direct relationships.

From the observation of the contour plots (Supplementary data, Section 3.1, Figure S5A–C), the smallest NLCs (Z-Ave = 148–128 nm, D50 = 46–56 nm, D90 = 110–160 nm) were found with the medium lipids ratio (7.94:1.94) and high surfactants ratio (4.5:0.5). These results can be explained by the fact that smaller particle sizes are generally obtained when higher concentrations of surfactants are used [46]. It has been described that higher surfactant concentrations ensure NLC stability, decreasing the surface tension and preventing aggregation and crystallization of lipids during storage, in turn avoiding the increased nanoparticle size [91].

#### 4.3.2. Effect of Lipid and Surfactant Ratios on PDI, ZP, and EE

The PDI is an indicative of the particle size distribution. According to the literature, a monodisperse sample has PDI values close to 0, while values between 0.1 and 0.3 indicate a narrow size distribution, between 0.1 and 0.4 indicate a moderate size distribution, and greater than 0.4 represent a wide size distribution [32,63]. From the analysis of the Pareto chart (Supplementary Data, Section 3.1, Figure S4D), the surfactants ratio (Tw/Ph) and lipids ratio (SL/LL) were not statistically significant for PDI, although the ratio of lipids showed a more significant effect than the surfactants ratio through the length of the bar. The lipids ratio (SL/LL) showed a linear and positive correlation with PDI, while the surfactants ratio had a negative effect, which means that PDI values are directly related to the lipids ratio. The contour plot (Supplementary Data, Section 3.1, Figure S5D) showed that the values of PDI were higher than 0.24 with the medium lipids ratio (7.94:1.94) and the high surfactants ratio (4.5:0.5), demonstrating a narrow size distribution for the NLC formulation.

ZP reflects the electric potential and the surface charge of nanoparticles in a suspension and is a predicting factor of the long-term stability. When ZP values are higher than │30│ mV, the electrostatic repulsion of the attractive Van der Waals forces stops nanoparticle aggregation from occurring [63,92,93,94]. Lipids arrangements on the surface of the nanoparticle, surfactant surface absorption, and the charge of the encapsulated drug interfere with ZP [32,92]. The Pareto chart showed that the lipids ratio had statistical significance (*p* = 0.05), showing a positive effect on ZP when a quadratic model was used, while the surfactant ratio had a significant negative effect on ZP for the quadratic model (Supplementary Data, Section 3.1, Figure S4E). Thus, ZP was more influenced by the lipids ratio than by the surfactants ratio. Regarding the ZP contour plot (Supplementary Data, Section 3.1, Figure S5E), optimum values were observed in the range of −32 mv and −34 mv, which were obtained with the medium lipids ratio (7.94:1.94) and high surfactants ratio (4.5:0.5).

The high negative ZP values were probably related to the co-surfactant and the drug. Phospholipon^®^ 90G is an amphoteric molecule that acquires a negative charge at the pH level of the NLC formulation, while rivastigmine is a Bronsted base, which when in aqueous dispersion forms a highly negatively charged hydroxyl group [78,95,96,97].

Regarding EE (Supplementary Data, Section 3.1, Figure S4F), only the lipids ratio (SL/LL) was statically significant (*p* = 0.05), although it negatively decreased as the lipids ratio increased. The contour plot (Supplementary Data, Section 3.1, Figure S5F) revealed that EE was higher than 95% when lipid and surfactant ratios were at medium levels (7.94:1.94 and 4.00:1.00, respectively). This can be explained by the lipophilic nature of rivastigmine, which becomes more solubilized as the lipid concentration increases. In addition, this can also be related to the surfactants, which create more available space between lipids to accommodate rivastigmine molecules [32,63].

From Table 4, it can be concluded that the CCD fitted to the selected CQAs, while the analysis of the Pareto charts (Supplementary Data, Section 3.1, Figure S4) and contour plots (Supplementary Data, Section 3.1, Figure S5) showed that the best lipids ratio was 7.94:1.94 (%, *w*/*w*) and the best surfactants ratio was 4.5:0.5 (%, *w*/*w*). Accordingly, this rivastigmine-loaded NLC formulation was selected for optimization of the instrumental parameters.

### 4.4. Part 2: Optimization of Instrumental Parameters by BBD

**Figure 2:**
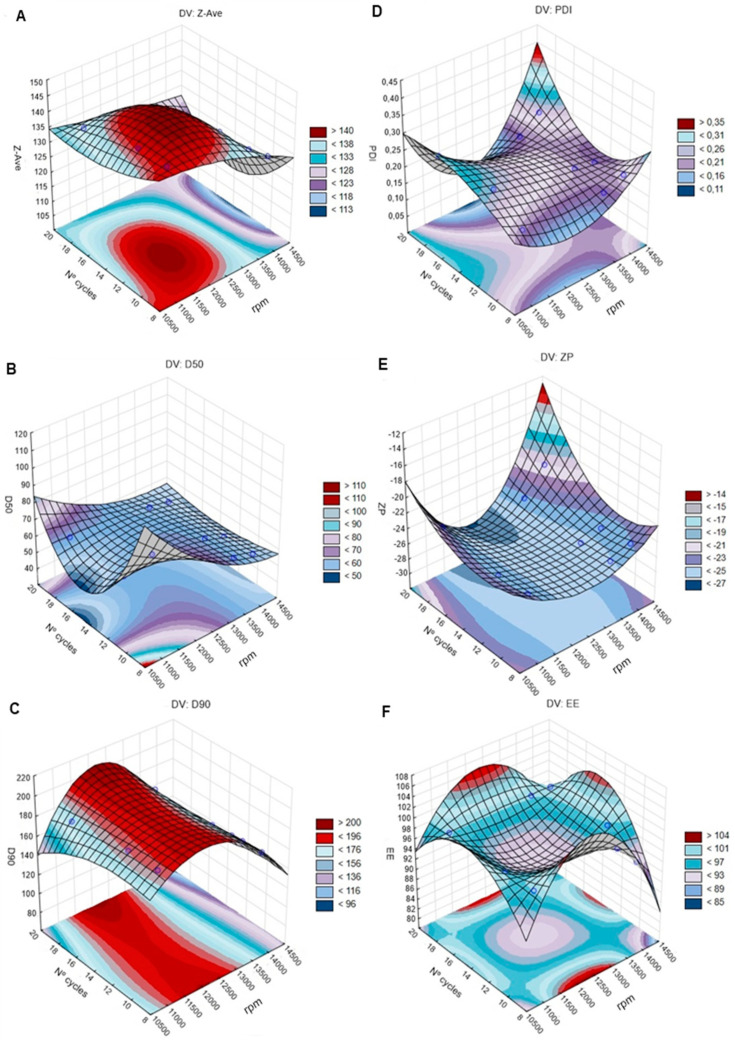
The 3-D surface plots portraying the effects of the number of high- pressure homogenization (HPH) cycles and emulsification speed (rpm) on the size (Z-Ave (mean particle size); D50 (50% of particles with size equal or lower to the given value) and D90 (90% of particles with size equal or lower to the given value)) (left: **A**–**C**), polydispersity index (PDI), zeta potential (ZP), and encapsulation efficiency (EE) (right: **D**–**F**).

**Figure 3:**
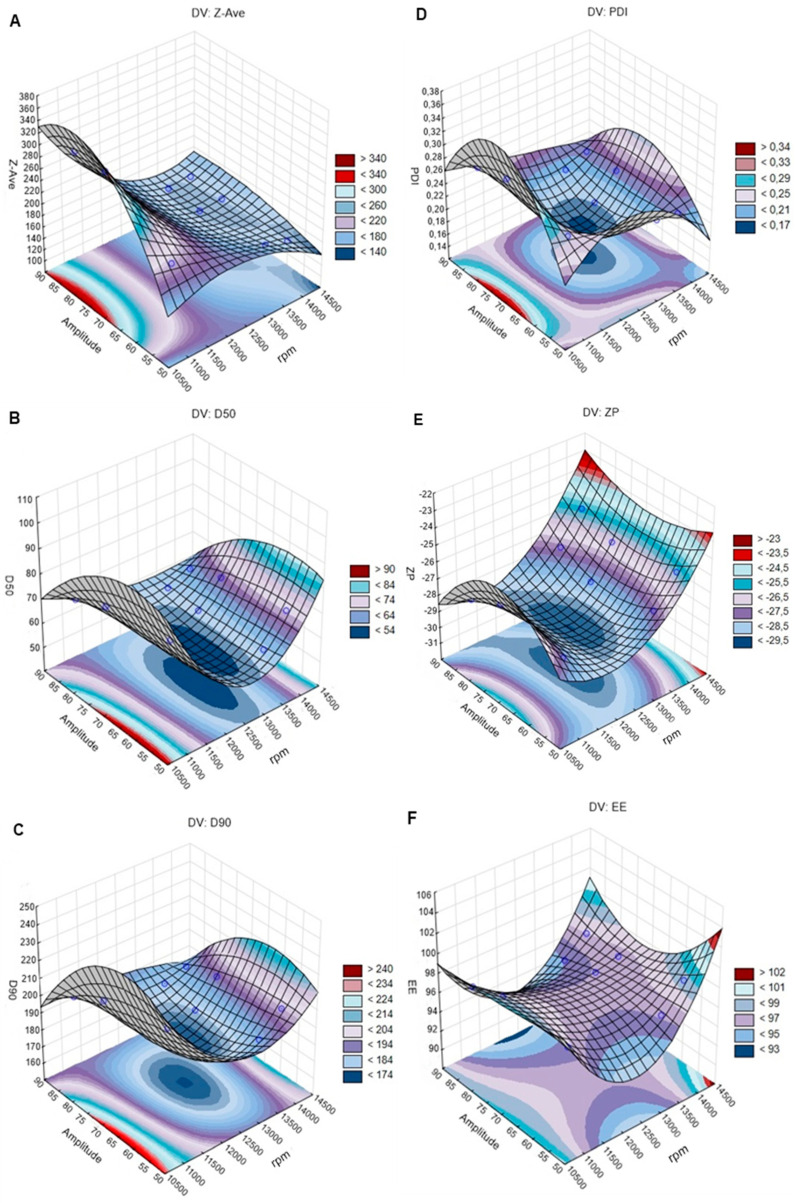
The 3-D surface plots portraying the effect of the sonication amplitude and emulsification speed (rpm) on the size (Z-Ave (mean particle size); D50 (50% of particles size equal or lower to the given value) and D90 (90% of particles with size equal or lower to the given value)) (left: **A**–**C**), polydispersity index (PDI), zeta potential (ZP), and encapsulation efficiency (EE) (right: **D**–**F**).

The effects of instrumental parameters (emulsification speed, sonication amplitude, and number of HPH cycles) on dependent responses were evaluated by means of a BBD. A value of *R*^2^ = 1 was obtained when 2-way interactions (linear quadratic) model was used (Supplementary Data, Section 3.2.1, Table S10 and Section 3.2.2, Table S11). Additionally, all variables were statistically significant (*p* = 0.001). Thus, the predicted and observed values were equal. The results are presented in 3-D response surface plots (Figure 2 and Figure 3).

#### 4.4.1. Effects of Emulsification Speed and HPH Cycles on Particles Size (Z-Ave, D50, and D90), PDI, ZP, and EE

##### Particle Size

The 3-D response surface plots for Z-Ave, D50, and D90 (Figure 2A–C) showed that as the emulsification speed (rpm) increased, the particle size decreased. In contrast, the number of HPH cycles did not cause a direct change in particle size, although a decrease was observed when the emulsification speed and the number of HPH cycles increased together.

The lowest and highest values of Z-Ave (124.80 and 141.90 nm, respectively), D50 (55.90 and 86.70 nm), and D90 (144.00 and 189.40 nm) were observed, respectively, when 14,000 rpm with 18 cycles and 11,000 rpm with 12 and 18 cycles were used. Therefore, it was concluded that higher emulsification speed and number of HPH cycles decrease the NLC size, which can be explained by the kinetic energy used in the high-speed emulsification process required to obtain a stable emulsion of uniform nanoparticle size [11]. In addition, the prolongation of the HPH process with more cycles promoted the breakdown of the emulsion oil droplets, since they suffered higher compression, turbulence, and cavitation within the homogenization gap [79,92,98]. Furthermore, the stability and bioavailability of the NLC dispersion were also improved [78,92]. Thus, 14,000 rpm with 18 homogenization cycles were set as the ideal conditions for the HPH method.

##### PDI, ZP, and EE

From the 3-D response surface plots (Figure 2D–F), it can be observed that as the emulsification speed and the number of HPH cycles change (increase or decrease), the values of PDI, ZP, and EE did not change significantly. PDI was around 0.21–0.26, indicating a narrow size distribution; ZP was in the range of −21 and −25 mV; EE ranged from 94% up to 98%. Thus, it was concluded that these parameters did not cause significant alterations in the rivastigmine-loaded NLC formulation.

#### 4.4.2. Effects of Ultrasound Technique on Particles Size (Z-Ave, D50, and D90), PDI, ZP, and EE

The selected design fitted to CQAs when the 2-way interaction (linear quadratic) model was used. The observed and predicted values for CQAs using the ultrasound technique were the same, namely *R*^2^ = 1 and *p* = 0.001 (Supplementary Data, Table S11).

##### Particle Size

The analysis of the 3-D response surface plots for Z-Ave, D50, and D90 (Figure 3A–C) revealed that as the emulsification speed increased, the particle size decreased. Regarding the sonication amplitude, a direct effect on particle size was not observed. However, when these parameters increased together, the particle size decreased. Z-Ave, D50, and D90 values were higher for 11,000 rpm and lower for 13,400 rpm. The increase of the emulsification speed allowed the formation of a stable rivastigmine-loaded NLC formulation with uniform particle size distribution [11]. Increasing the sonication amplitude results in higher ultrasonic wave energy with a consequent increase in the shear cavitation forces, leading to the breakdown of the emulsion oil droplets to nanometric sizes [46,51].

The lowest and highest Z-Ave (295 and 152 nm), D50 (57 and 80 nm), and D90 (180 and 220 nm) values were observed when 13,400 rpm and 85% amplitude were applied. Thus, 13,400 rpm with 85% amplitude were set as the desired conditions for the ultrasound technique.

##### PDI, ZP, and EE

The results showed that the values obtained for PDI, ZP, and EE (Figure 3D–F) did not change significantly with variations of the emulsification speed and sonication amplitude, as observed for the HPH method. PDI values were between 0.21 and 0.25, while ZP ranged between −26 mV and −28 mV. The EE values ranged from 95% up to 97%, being close to 99% for 14,000 rpm with 55% amplitude. The small differences in ZP and EE values can be attributed to the different locations of the anionic drug molecules in the lipid matrix [32,46].

### 4.5. Model Validation

Two rivastigmine-loaded NLC formulations with the selected ratios of lipids and surfactants were produced under the most suitable instrumental parameters found for the ultrasound technique and the HPH method. The results of the observed responses were within the design space and close to the predicted values, which allowed for model validation (Table 5).

Regarding the observed responses in Table 5, other studies involving drug-loaded NLCs for nose-to-brain delivery have reported similar values for ZP, particle size, PDI, and EE. For example, Jain et al. optimized an artemether-loaded NLC for intranasal delivery with a particle size of 123.4 nm, ZP of −34.4 mV, and EE of 91.2% [99]. Gadhave et al. developed a teriflunomide-loaded NLC with a particle size of 99.8 nm, PDI of 0.35, ZP of −22.3 mV, and EE of 83.4% [31]. Madane and Mahajan prepared a curcumin-loaded NLC with a particle size of 146.8 nm, PDI of 0.18, EE of 90.86%, and ZP of −21.4 mV [33].

### 4.6. pH and Osmolarity

The pH of the optimized rivastigmine-loaded NLC formulations produced by ultrasound technique and HPH method were adjusted to the nasal mucosa values (5.5–6.6) with a dilute HCl solution. Similarly, the osmolarity was adjusted with glycerin to the physiological range of 230–320 mOsm/kg, obtaining isotonic formulations compatible with the nasal mucosa [100]. The final values for the pH and osmolarity of the optimized rivastigmine-loaded NLC formulations were 6.22 ± 0.01 and 280 ± 1 mOsm/Kg for the ones produced by HPH method; and 6.21 ± 0.01 and 279 ± 1 for the ones produced by ultrasound technique. Furthermore, it was confirmed that the CQAs values were not altered after addition of HCl and glycerin (Supplementary Data, Section 4, Table S12).

### 4.7. In Vitro Drug Release Studies

**Figure 4:**
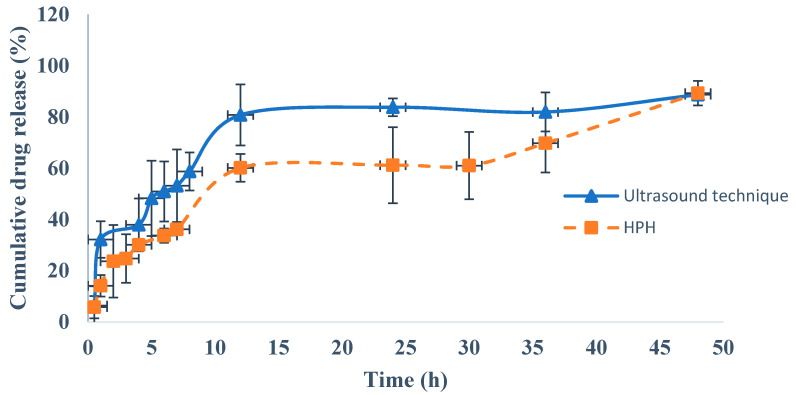
Cumulative percentage of drug release in phosphate-buffered solution (pH 6.4) from rivastigmine-loaded nanostructured lipid carriers (NLC) produced by ultrasound technique and rivastigmine-loaded NLC produced by high-pressure homogenization (HPH) method.

**Figure 5:**
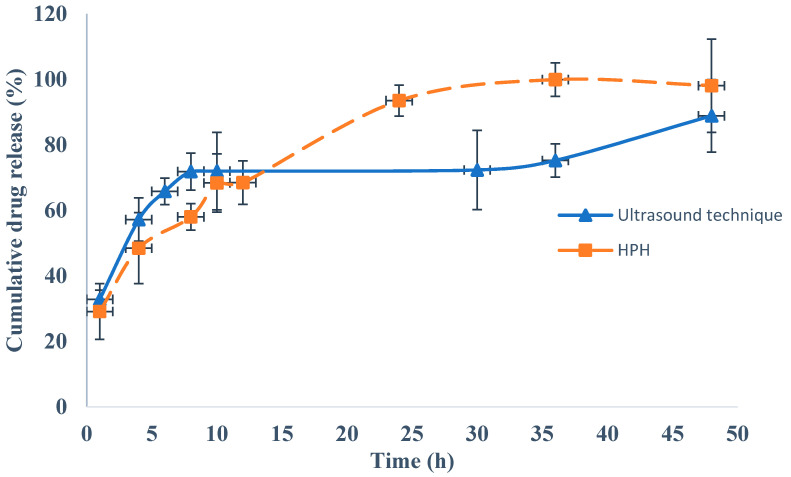
Cumulative percentage of drug release in simulated nasal electrolyte solution (pH 6.4) from rivastigmine-loaded nanostructured lipid carriers (NLC) produced by ultrasound technique and rivastigmine-loaded NLC produced by high-pressure homogenization (HPH) method.

The release profile of rivastigmine from the NLC produced by HPH method and ultrasound technique was assessed in phosphate-buffered solution at pH 6.4 (Figure 4) and in simulated nasal electrolyte solution at pH 6.4 (Figure 5) over a period of 48 h.

From Figure 4, an initial fast drug release can be observed from both rivastigmine-loaded NLC formulations, which is related to the drug diffusion from the surface of the NLC to the dissolution medium, followed by a prolonged release [15,98]. This phenomenon can be explained by the rapid solidification of the solid lipids during the formation of the NLC, which results in nanoparticles with an internal core containing a low amount of liquid lipids, which in turn accumulate in the outermost layers. As rivastigmine is an oil, it also tends to accumulate on the surface of the NLC, meaning it is released more quickly [33,99,101].

For both optimized NLC formulations, from 4 up to 48 h, the release of rivastigmine was controlled by the diffusion rate of the drug through the lipid matrix or by the lipid matrix degradation in the dissolution medium [62,102]. For the rivastigmine-loaded NLC produced by the ultrasound technique, about 80.75 ± 7.43% of rivastigmine was released after 12 h and the maximum drug release (88.67 ± 3.45%) was observed at 48 h. In contrast, for the rivastigmine-loaded NLC produced by the HPH method, at 12 h the release of rivastigmine was lower (60.13 ± 3.12%) and the maximum drug release (89.25 ± 3.22%) was observed at 48h. Statistically significant differences between the rivastigmine-loaded NLC produced by ultrasound technique and rivastigmine-loaded NLC produced by HPH method were observed (*p* < 0.05).

Figure 5 shows that in the simulated nasal electrolyte solution, similarly to the phosphate-buffered medium (Figure 4), an initial fast drug release was observed for both rivastigmine-loaded NLC formulations, followed by a prolonged release. The release rate of rivastigmine was higher for the rivastigmine-loaded NLC produced by the HPH method in the first 15 h when compared to the one produced by ultrasound technique, and the process reverted from the 15 h up to 48 h. Nonetheless, for both formulations a sustained drug release effect was observed, showing that the drug molecules were entrapped within the lipid matrix and that there was a homogeneous distribution of the liquid lipid droplets in the solid lipids of the NLC, as described in other research studies [29,50,103]. For the rivastigmine-loaded NLC produced by ultrasound technique, a maximum drug release of 88.90 ± 8.42% was obtained at 48 h, whereas for the rivastigmine-loaded NLC produced by the HPH method the maximum drug release was 98.10 ± 7.98% at 48 h. Statistically significant differences (*p* < 0.05) between the rivastigmine-loaded NLC produced by ultrasound technique and the rivastigmine-loaded NLC produced by HPH method were observed.

Therefore, a slower release of the drug was observed for the rivastigmine-loaded NLC formulation prepared by the ultrasound technique in the two dissolution media tested.

After fitting the in vitro release results of the two tested dissolution media to the kinetic models, it was observed that the Korsmeyer–Peppas model presented the highest *R*^2^ values (0.9780 and 0.9848 for phosphate-buffered solution at pH 6.4 and simulated nasal electrolyte solution, respectively) (Table 6). An *n* value between 0.599 and 0.670 indicated that the drug release follows an anomalous transport route, i.e., a combination of non-Fickian release and Fickian release, which can be explained by the initial fast release of rivastigmine followed by prolonged release, indicating a biphasic behavior. Other authors have reported similar results for in vitro drug release from NLCs across dialysis membranes. For instance, the release of teriflunomide from a NLC intranasal formulation in simulated nasal electrolyte solution followed a biphasic behavior, with 75.11% of the drug being released after 8 h. Similar values were observed for the optimized rivastigmine-loaded NLC produced by ultrasound technique (75.89%) after the same period of time and using the same dissolution media (Figure 5) [31]. Jazuli et al. conducted drug release studies with lurasidone-loaded NLCs for nose-to-brain delivery and observed fast drug release after 12 h followed by sustained drug release, with a maximum release of 92.12% after 24 h [101]. This biphasic behavior was also observed with both optimized rivastigmine-loaded NLC formulations (Figure 4 and Figure 5), although the maximum drug release was observed after 48 h. Alam et al. observed sustained in vitro release of isradipine from a NLC, with a maximum value of 92.89% after 24 h [46], which was also observed for the rivastigmine release from the optimized NLC produced by HPH (93.55%) after 24 h (Figure 5). Garg et al. studied the in vitro release profile of thirteen aceclofenac-loaded NLC formulations [104] and observed similar patterns of biphasic drug release, with a maximum release of around 80% after 48 h. Similar patterns were observed for the optimized rivastigmine-loaded NLC prepared by ultrasound technique (88.67%) and by HPH (89.25%) (Figure 4).

In vitro drug release studies are routinely employed during the optimization of NLC formulations. However, it is important to keep in mind that these studies are limited as a means of evaluating the in vivo performance of NLC formulations. Therefore, experiments evaluating the in vitro biocompatibility in nasal cell culture models and ex vivo studies in nasal mucosa must be carried out to obtain information about the toxicity, permeability, and transport of the optimized rivastigmine-loaded NLC formulation in the nasal mucosa. In addition, in vivo tests on animals should be performed to confirm the effectiveness of this formulation for the direct delivery of rivastigmine from the nose to the brain.

### 4.8. Stability Studies

Table 7 shows the results of stability studies of optimized rivastigmine-loaded NLC formulations, where it can be seen that although the NLC exhibited sustained drug release (Section 4.7), after 90 days of storage at different temperatures the particle size, PDI, and ZP values showed slight increases, while the EE value showed a slight decrease. These results suggest that both optimized rivastigmine-loaded NLC formulations are stable during storage and fulfil the QTPP for nasal administration. This high stability is related to the presence of Tween^®^ 80 and Phospholipon^®^ 90 G, which stabilize NLC via distinct mechanisms (steric and electrostatic, respectively) and also to the presence of vitamin E, which has antioxidant activity that provides chemical stability to rivastigmine and prevents oxidation of the lipid matrix [31,34,56,57,63]. Besides, it has been described that NLC formulations with ZP values close to |30| mV show high long-term stability [105]. Nonetheless, stability studies should be performed for longer periods to confirm these data.

Some authors have reported similar results for the long-term stability of NLC formulations. For example, Huang et al. developed three NLC formulations containing co-encapsulated quercetin and linseed oil, which showed high stability over 90 days of storage at 25 °C. The initial particle size values were 89.2, 91.3, and 95.6 nm, while the initial EE values were 95.9%, 94.5%, and 93.6%. After 90 days, small increases in the particle size (<7 nm) and EE (<3%) were observed. In addition, the NLC co-encapsulated with quercetin and linseed oil showed sustained drug release [106]. Gadhave et al. developed a NLC formulation for intranasal delivery of teriflunomide, with a particle size of 99.82 nm, ZP of −22.29 mV, and EE of 83.39%. Accelerated stability studies performed over 6 months at 40 °C and 75% relative humidity showed that the evaluated parameters were within acceptable limits, without suffering significant changes, indicating the good stability of the NLC formulation. In addition, teriflunomide-loaded NLC also showed a sustained drug release profile [31]. Garg et al. carried out stability studies on an optimized aceclofenac-loaded NLC formulation over 90 days of storage. On the production day, the aceclofenac-loaded NLC showed a particle size of 230 nm and PDI of 0.16. After storage at three different temperatures (2–8 °C, 2 °C and 60% relative humidity, and 40 °C and 75% relative humidity), no significant changes were observed in these values, with the respective particle sizes being 228.3, 239.8, and 251.1 nm; and with respective PDI values of 0.21, 0.27, and 0.33. This NLC formulation also showed sustained release of about 80% aceclofenac after 48 h [104]. Cavalcanti et al. optimized a zidovudine-loaded NLC, which showed high stability and sustained drug release. The optimized formulation had a particle size of 266 nm, PDI of 0.168, and ZP of −29 mV. After 45 days of storage at 4 °C, the formulations maintained their physical stability, without showing significant changes. Additionally, in vitro release studies showed 100% drug release from the NLC after 45 h [107]. Jojo et al. prepared a NLC formulation for intranasal delivery of pioglitazone, which had a particle size of 211.4 nm, PDI of 0.257, ZP of 14.9 mV, and EE of 70.18%. Stability studies were performed for 90 days at 4 and 25 °C, and no significant changes were observed in the investigated parameters. In addition, the pioglitazone-loaded NLC also showed a biphasic pattern, with an initial fast drug release followed by sustained drug release, reaching about 50% release after 24 h [108].

## 5. Conclusions

A rivastigmine-loaded NLC formulation was successfully optimized using the QbD approach and other tools, namely an Ishikawa diagram, DoE, Pareto chart, and response surface plots. The developed HPLC method was found to be simple, linear, precise, selective, and robust for rivastigmine quantification. Regarding the optimization of the rivastigmine-loaded NLC formulation, the central composite design used to select the best ratios of lipids and surfactants and the Box–Behnken design used to obtain the best instrumental parameters were considered suitable and statistically significant for the CQAs, providing the selection of the most suitable formulations with a 95% confidence level.

The optimized rivastigmine-loaded NLC formulation had a solid lipid/liquid lipid ratio of 7.94:1.94 (%, *w*/*w*) and a surfactant/co-surfactant ratio of 4.5:0.5 (%, *w*/*w*). Regarding the production methods, the most adequate conditions were an emulsification rate of 13,400 rpm with 85% sonication amplitude for the ultrasound technique and an emulsification rate of 14,000 rpm with 18 cycles for the HPH method. The latter was considered the most suitable method to prepare the rivastigmine-loaded NLC formulation with the desirable CQAs, although the ultrasound technique also showed effectiveness.

The results showed that the optimized formulations produced by ultrasound technique and HPH method presented respective particle sizes of 114.0 ± 1.9 nm and 109.0 ± 0.9 nm, PDI values of 0.221 ± 0.003 and 0.196 ± 0.007, ZP values of −30.6 ± 0.3 mV and −30.5 ± 0.3 mV, and EE values of 97.0 ± 0.5% and 97.2 ± 0.3%. In addition, no significant changes in these CQAs were observed after 90 days of storage at different temperatures. In vitro studies showed the achievement of a biphasic release profile, resulting from the occurrence of an initial fast release followed by prolonged release of rivastigmine from the NLC formulations produced by both techniques over 48 h.

The results of our study suggest that the optimized rivastigmine-loaded NLC formulation produced by the HPH method is stable and can be used as an alternative delivery system for the nose-to-brain delivery of rivastigmine. However, this application must be confirmed with more in vitro and in vivo animal experiments before reaching clinical studies. In addition, QbD has proven to be a very useful approach for the optimization of NLC formulations with specific requisites.

## Figures and Tables

**Table 1:** Design of experiment (DoE) using six central composite design (CCD) for rivastigmine-loaded nanostructured lipid carriers (NLC) formulations with different critical material attributes (CMAs).

Formulation Variables		Levels		
X1 Precirol^®^ ATO 5: Vitamin E ratio (%, *w*/*w*) X2 Tween^®^ 80: Phospholipon^®^ 90G concentration (%, *w*/*w*)	X1 X2	**Low (−1)**	**Medium (0)**	**High (+1)**
		5.94:3.94	6.94:2.94	7.94:1.94
		2.00:1.00	2.50:0.50	2.50:1.50
	X1 X2	6.94:2.94	7.94:1.94	8.94:0.94
		2.00:1.00	2.50:0.50	2.50:1.50
	X1 X2	5.94:3.94	6.94:2.94	7.94:1.94
		3.00:1.00	3.50:0.50	3.50:1.50
	X1 X2	6.94:2.94	7.94:1.94	8.94:0.94
		3.00:1.00	3.50:0.50	3.50:1.50
	X1 X2	5.94:3.94	6.94:2.94	7.94:1.94
		4.00:0.40	4.00:1.00	4.50:0.50
	X1 X2	6.94:2.94	7.94:1.94	8.94:0.94
		4.00:0.40	4.00:1.00	4.50:0.50

**Table 2:** Design of experiment (DoE) using Box–Behnken design (BBD) to optimize rivastigmine-loaded nanostructured lipid carriers (NLC) formulations using different combinations of critical process parameters (CPPs).

Instrumental Parameters		Levels		
		Low (−1)	Medium (0)	High (+1)
X1: Emulsification speed (rpm) + X2: HPH cycles	X1	11,000	13,400	14,000
	X2	9	12	18
X1: Emulsification speed (rpm) + X3: sonication amplitude	X1	11,000	13,400	14,000
	X3	55	75	85

**Table 3:** Observed (O) and predicted (P) results for critical quality attributes (CQAs).

**Critical Quality Attributes (CQAs)**	Z-Ave (nm) ^1^		D50 (nm) ^2^		D90 (nm) ^2^		PDI ^3^		ZP ^4^ (mV)		EE ^5^ (%)	
Runs	O ^6^	P ^7^	O ^6^	P ^7^	O ^6^	P ^7^	O ^6^	P ^7^	O ^6^	P ^7^	O ^6^	P ^7^
1	166.600 ± 1.911	175.000	58.400 ± 0.102	58.650	148.602 ± 0.570	163.740	0.221 ± 0.003	0.224	−28.000 ± 0.253	−28.900	94.001 ± 0.143	94.690
2	158.301 ± 0.852	150.450	61.401 ± 0.244	57.950	159.600 ± 0.992	155.040	0.224 ± 0.007	0.230	−28.600 ± 0.251	−29.020	94.890 ± 0.271	94.340
3	187.104 ± 0.980	190.190	51.100 ± 0.132	53.440	148.603 ± 0.793	161.200	0.263 ± 0.002	0.247	−29.000 ± 0.192	−29.670	92.900 ± 0.232	93.810
4	173.305 ± 1.231	160.140	75.900 ± 0.140	74.540	256.002 ± 0.651	248.900	0.234 ± 0.004	0.230	−33.300 ± 0.231	−33.490	93.594 ± 0.181	93.260
5	166.600 ± 0.893	165.220	59.901 ± 0.190	61.920	182.001 ± 0.733	176.180	0.225 ± 0.001	0.212	−27.600 ± 0.280	−26.890	93.761 ± 0.310	93.730
6	176.701 ± 0.972	182.820	70.900 ± 0.171	69.970	243.000 ± 0.651	240.760	0.224 ± 0.003	0.227	−31.000 ± 0.300	−30.600	92.702 ± 0.251	92.350
7	192.303 ± 0.114	183.180	51.200 ± 0.150	49.130	146.000 ± 0.910	128.040	0.242 ± 0.004	0.250	−31.300 ± 0.371	−30.400	96.390 ± 0.401	95.320
8	130.700 ± 0.791	144.560	60.401 ± 0.221	63.560	174.001 ± 0.882	183.900	0.251 ± 0.001	0.242	−33.400 ± 0.193	−33.190	94.001 ± 0.254	94.690
9	192.300 ± 0.150	192.300	61.301 ± 0.143	61.300	207.000 ± 0.980	207.000	0.243 ± 0.002	0.245	−32.300 ± 0.204	−32.300	95.500 ± 0.190	95.500
10	192.300 ± 0.150	192.300	61.301 ± 0.143	61.300	207.000 ± 0.980	207.000	0.234 ± 0.002	0.245	−32.300 ± 0.204	−32.300	95.500 ± 0.190	95.500

^1^ Z-Ave (mean particle size); ^2^ volume distribution (50% of particles with size equal or lower to the given value of D50) and 90% of particles with size equal or lower to the given value of D90)); ^3^ PDI (polydispersity index); ^4^ ZP (zeta potential); ^5^ EE (encapsulation efficiency) ^6^ O (observed results); ^7^ P (predicted results).

**Table 4:** Results of particle size (Z-Ave (mean particle size); volume distribution (50% of particles with size equal or lower to the given value of D50 and 90% of particles with diameter equal or lower to the given value of D90), polydispersity index (PDI), zeta potential (ZP), and encapsulation efficiency (EE) tests for the rivastigmine-loaded nanostructured lipid carriers (NLC) selected following the optimization of formulation variables.

Tested Ratios (*w*/*w*, %)		DoE ^1^		LD ^2^ (Mean ± SD ^3^, *n* ^4^ = 5)		DLS ^5^ (Mean ± SD ^3^, *n* ^4^ = 5)			
SL/LL ^6^	Tw/Ph ^7^	Levels		D50 ^8^ (nm)	D90 ^9^ (nm)	Z-Ave ^10^ (nm)	PDI ^11^	ZP ^12^ (mV)	EE ^13^ (%)
5.94:3.94	4.0:0.4	−1.00	−1.00	58.402 ± 0.009	148.600 ± 0.009	166.602 ± 0.010	0.221 ± 0.011	−28.000 ± 0.011	94.001 ± 0.012
5.94:3.94	4.5:0.5	−1.00	1.00	61.400 ± 0.008	159.603 ± 0.010	158.300 ± 0.008	0.213 ± 0.010	−28.601 ± 0.010	94.890 ± 0.008
7.94:1.94	4.0:0.4	1.00	−1.00	51.101 ± 0.010	148.604 ± 0.011	187.101 ± 0.012	0.251 ± 0.010	−29.000 ± 0.008	92.903 ± 0.011
7.94:1.94	4.5:0.5	1.00	1.00	75.903 ± 0.008	256.012 ± 0.008	173.302 ± 0.013	0.231 ± 0.009	−33.300 ± 0.011	93.590 ± 0.013
5.94:3.94	4.0:1.0	−1.41	0	59.902 ± 0.010	182.013 ± 0.007	166.603 ± 0.009	0.220 ± 0.010	−27.600 ± 0.010	93.760 ± 0.007
6.94:2.94	4.5:0.5	0	1.41	60.401 ± 0.011	174.020 ± 0.011	130.703 ± 0.011	0.251 ± 0.011	−33.400 ± 0.009	94.001 ± 0.010
8.94:0.94	4.5:0.5	1.00	1.00	63.200 ± 0.010	199.211 ± 0.010	174.200 ± 0.009	0.290 ± 0.010	−32.900 ± 0.010	98.300 ± 0.011

^1^ DoE (design of experiment); ^2^ LD (laser diffraction); ^3^ SD (standard deviation); ^4^n (number of runs); ^5^ DLS (dynamic light scattering); ^6^ SL/LL (solid lipid: liquid lipid); ^7^ Tw/Ph (Tween^®^ 80: Phospholipon^®^ 90G); ^8^ D50 (50% of particles with a diameter size equal or lower to the given values); ^9^ D90 (90% of particles with a diameter size equal or lower to the given values); ^10^ Z-Ave (mean particle size); ^11^ PDI (polydispersity index); ^12^ ZP (zeta potential); ^13^ EE (encapsulation efficiency).

**Table 5:** Observed and predicted response values of the two optimized rivastigmine-loaded nanostructured lipid carriers (NLC) formulations.

**Observed Responses**	**Ultrasound Technique**	**High-Pressure Homogenization (HPH) Method**
Z-Ave ^1^ (nm)	114.000 ± 1.910	109.000 ± 0.850
PDI ^2^	0.221 ± 0.003	0.196 ± 0.007
ZP ^3^ (mV)	−30.633 ± 0.288	−30.466 ± 0.252
EE ^4^ (%)	96.987 ± 0.446	97.174 ± 0.297
**Predicted Responses**	**Ultrasound Technique**	**High-Pressure Homogenization (HPH) Method**
Z-Ave ^1^ (nm)	155.000	124.000
PDI ^2^	0.190	0.242
ZP ^3^ (mV)	−28.400	−29.100
EE ^4^ (%)	95.140	97.600

Results presented as mean ± SD (*n* = 3); ^1^ Z-Ave: mean particle size; ^2^ PDI: polydispersity index; ^3^ ZP: zeta potential; ^4^ EE: encapsulation efficiency.

**Table 6:** Results of the curve fitting into different kinetic models for rivastigmine-loaded nanostructured lipid carriers (NLC) formulations prepared by ultrasound technique and high-pressure homogenization (HPH) method.

Release Media	Formulation	*R* ^2^				*n*
		Zero Order	First Order	Higuchi Model	Korsmeyer–Peppas	
PBS, pH 6.4	NLC_s_	0.649	0.796	0.799	0.936	0.636
	NLC_HPH_	0.773	0.796	0.919	0.978	0.670
SNE, pH 6.4	NLC_s_	0.630	0.785	0.757	0.978	0.599
	NLC_HPH_	0.859	0.613	0.954	0.985	0.667

NLC_S_: rivastigmine-loaded NLC produced by ultrasound technique; NLC_HPH_: rivastigmine-loaded NLC produced by HPH; PBS: phosphate-buffered solution; SNE: simulated nasal electrolyte solution; *R*^2^: correlation coefficient.

**Table 7:** Results of the stability studies of rivastigmine-loaded nanostructured lipid carriers (NLC) formulations prepared by ultrasound technique and high-pressure homogenization (HPH) method.

Formulation	Day	T ^1^ (°C)	D50 ^2^ (nm)	D90 ^2^ (nm)	Z-Ave ^3^ (nm)	PDI ^4^	ZP ^5^ (mV)	EE ^6^ (%)
**NLC_s_**	0	-	57.972 ± 0.971	184.300 ± 0.721	114.094 ± 0.990	0.221 ± 0.003	−30.610 ± 0.321	96.983 ± 0.421
	90	4.0 ± 0.5	60.590 ± 0.574	189.981 ± 0.995	116.230 ± 0.911	0.224 ± 0.020	−30.901 ± 0.452	94.580 ± 0.111
		20.0 ± 0.5	67.653 ± 0.750	200.760 ± 0.651	125.630 ± 0.764	0.227 ± 0.005	−31.073 ± 0.694	94.677 ± 0.140
**NLC_HPH_**	0	-	55.971 ± 0.831	144.322 ± 0.972	109.400 ± 0.895	0.196 ± 0.007	−30.470 ± 0.394	97.152 ± 0.341
	90	4.0 ± 0.5	65.293 ± 0.654	199.674 ± 0.913	111.780 ± 0.001	0.212 ± 0.004	−29.971 ± 0.410	95.416 ± 0.980
		20.0 ± 0.5	68.890 ± 0.543	211.763 ± 0.742	114.980 ± 0.852	0.210 ± 0.003	−30.050 ± 0.540	94.448 ± 0.991

Results are presented as mean ± SD (*n* = 3); NLC_S_: rivastigmine-loaded NLC produced by ultrasound technique; NLC_HPH_: rivastigmine-loaded NLC produced by HPH; ^1^ Temperature; ^2^ Volume distribution: D50 and D90; ^3^ Z-Ave: mean particle size; ^4^ PDI: polydispersity index; ^5^ ZP: zeta potential; ^6^ EE: encapsulation efficiency.

## Supplementary Materials

The following are available online at https://www.mdpi.com/1999-4923/12/7/599/s1, Figure S1. Calibration plot of areas (mean) *versus* rivastigmine concentration (*n* = 3). Figure S2. Chromatogram of the supernatant of placebo-NLC formulation. Figure S3. Chromatogram of standard 1200 µg/mL rivastigmine solution. Figure S4. Pareto chart showing the effects of CMAs on CQAs, *viz*., size (Z-Ave, D50 and D90) (left: A-C), PDI, ZP and EE (right: D-F). Figure S5. Contour plot for CQAs, *viz*., size (Z-Ave, D50 and D90) (left: A-C); and PDI, ZP and EE (right: D-F). Figure S6. Filter paper showing the results of screening of drugs and lipids, where the absence of oil droplets resulting from the solubilisation of drug in the lipid mixture is observed. Table S1: System suitability parameters. Table S2. Results achieved for the intra-day precision and inter-day precision. Table S3: Results obtained for the instrumental precision. Table S4. Drug recovery for method accuracy. Table S5: Detection and quantification limits. Table S6. Results of the method robustness after variation the flow rate. Table S7: Results of the method robustness after variations in the mobile phase. Table S8: Results of encapsulation efficiency (EE) and loading capacity (LC) of rivastigmine-loaded NLC formulations. Table S9: ANOVA models and respective R squared (R^2^). Table S10: ANOVA models and respective *R*^2^ for instrumental parameters: emulsification speed and number of HPH cycles. Table S11. ANOVA models and respective R^2^ for instrumental parameter: sonication amplitude. Table S12: Critical quality attributes (CQAs) values of rivastigmine-loaded NLC formulations before and after the pH and osmolarity adjustment by addition of HCl and glycerin.
