# Supplementary material for: Double Optimization of Rivastigmine-Loaded Nanostructured Lipid Carriers (NLC) for Nose-to-Brain Delivery Using the Quality by Design (QbD) Approach: Formulation Variables and Instrumental Parameters
Source: Pharmaceutics. 2020 Jun 28;12(7):599. doi: 10.3390/pharmaceutics12070599 (PMC7407548; doi:10.3390/pharmaceutics12070599)
Supplement: Supplementary file 1 [file pharmaceutics-12-00599-s001.pdf]

pharmaceutics

Supplementary Materials: Double Optimization of Rivastigmine-Loaded Nanostructured Lipid Carriers (NLC) for Nose-to-Brain Delivery Using the Quality by Design (QbD) Approach: Formulation Variables and Instrumental Parameters

Sara Cunha, Cláudia Pina Costa, Joana A. Loureiro, Jorge Alves, Andreia F. Peixoto, Ben Forbes, José Manuel Sousa Lobo and Ana C. Silva

1. HPLC Method Validaton

Method validation was performed according to the International Conference on Harmonization (ICH) guidelines [1–3]:

1.1. System Suitability

This parameter is used to analyse the method as an integral system and ensures that the developed method can produce acceptable results, based on the concept of equipment, analytical procedures and analyse of samples. System suitability was evaluated through the results of repeated injection (*n* = 10) of rivastigmine standard solution (1200 µg/mL). The calculated parameters were number of theoretical plates (N), retention time (t_R), retention factor (K') and asymmetry (As).

1.2. Linearity

Linearity is the method ability to achieve results directly proportional to the concentration of the analyte in the sample and was accessed using six dilutions (24, 48, 72, 120 and 840 µg/mL) of a standard stock solution with 1200 µg/mL of rivastigmine. A calibration curve was constructed, and a linearity correlation was established. The corresponding regression plot demonstrated a nearly perfect linear relation (R^2 = 0.999) over the concentration range of 24–1200 µg/ml that covered the concentrations of rivastigmine standard solutions.

1.3. Precision

Method precision is the agreement between the results obtained for a series of measurements of distinct equivalent samples and can be assessed by repeatability or intra-day precision and intermediate precision or inter-day precision, expressed as relative standard deviation (RSD, %). An investigation of precision was done using six standard solutions to calculate the RSD for three standard solutions with low, medium and high concentrations (48, 120, 1200 µg/ml, respectively). Intra-day precision was determined by analysing six replicates of three different rivastigmine concentrations on the same day. The results were evaluated in terms of RSD with an acceptance limit for rivastigmine solutions below 1%. Inter-day precision was assessed using nine replicates of three different rivastigmine concentrations in three different days. The results were evaluated in terms of RSD with an acceptance limit for rivastigmine solutions below 1%. Instrumental precision was also assessed by performing, in the same day, ten injections of a high concentration standard solution (1200 µg/mL) and three samples of supernatant of rivastigmine-loaded NLC formulation.

1.4. Accuracy

Accuracy was used to express the closeness of agreement between the value found and an accepted reference value. It determines if the method can accurately quantify the drug in the presence of other compounds, such as reactions components, excipients and release medium, and can be assessed after establish method linearity, precision and specificity. Three different concentrations of

rivastigmine standard solutions (48, 120 and 1200 μg/mL) were analysed in triplicate and the results were calculated in comparison to the amount of rivastigmine added with the obtained by the method, considering that rivastigmine purity was 99.9%, as specified by the manufacturer. Method range was selected from linearity, precision and accuracy tests.

1.5. Specificity

Specificity evaluates the ability of the method to analyse the interference of matrix components (lipids and surfactants) and degradation products in the analyte quantification. The selectivity of the method was evaluated using drug-free NLC supernatant formulations and comparing the chromatogram obtained with the one of 1200 μg/mL rivastigmine standard.

1.6. Detection limit (DL)

DL is defined as the minimum amount of analyte that could be detected but not necessarily quantified as an exact value and was calculated using the calibration curve and applying the following equation: $DL = 3.3\ \sigma/S$, where σ is the standard deviation of the response and S the slope of the curve.

1.7. Quantification Limit (QL)

QL is the smallest possible quantity of analyte in a sample that could be determined with precision and accuracy. The QL could be assessed by the calibration curve and applying the equation: $QL=10\sigma/S$, where σ is the standard deviation of the response and S the slope of the curve.

1.8. Robustness

Robustness is the ability of the method to stay qualitatively and quantitatively stable after small deliberate variations of the chromatographic experimental conditions, indicating the reliability during procedures. Method robustness was assessed by evaluating the effects on the peak retention time, recovery percentage and RSD values, obtained after undergoing slight variations in the mobile phase concentration and in the flow rate.

2. Results

2.1. System Suitability

Table S1. System suitability parameters.

Parameters[a]		Acceptance limits [4-7]
**Number of theoretical plates (N)**	2841.896 ± 42.660	> 2000
**Retention time (t_R)**	1.513 ± 0.001	-
**Retention factor (K′)**	0.513 ± 0.001	> 2
**Peak asymmetry factor (As)**	0.795 ± 0.005	0.8-1.2 Good<br>1.2-2.0 Marginal<br>≥ 2.0 Unacceptable

[a] Mean value ± standard deviation.

2.2. Linearity

A linearity standard calibration curve was obtained over the concentration range of 24-1200 μg/mL (Figure S1).

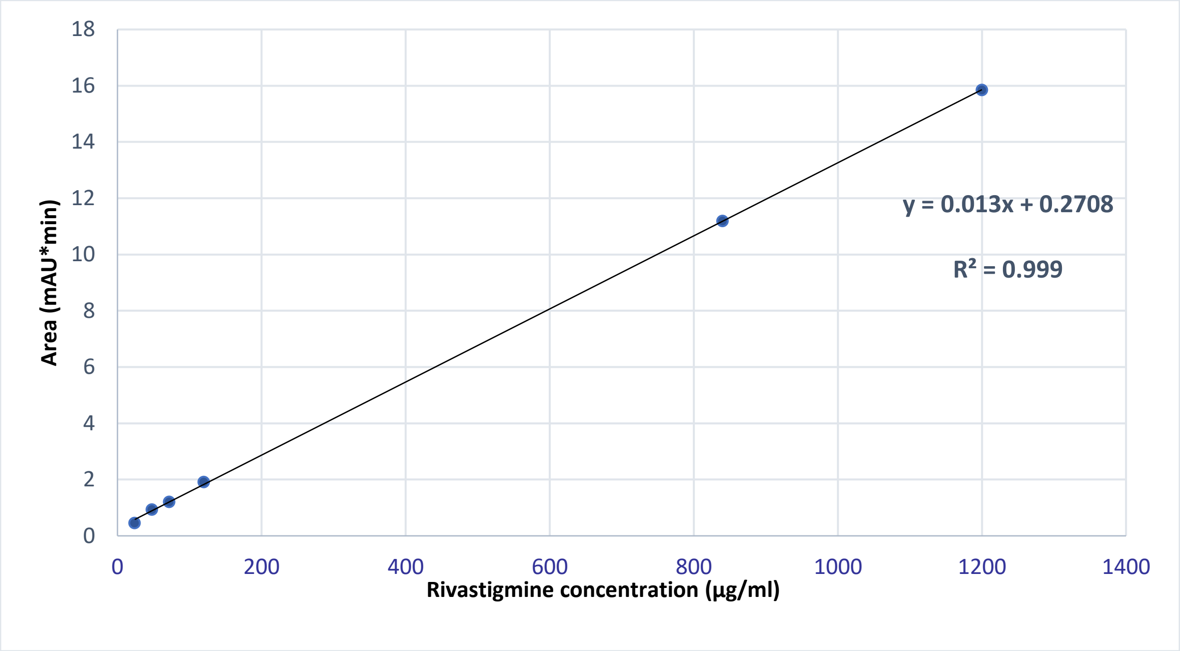

Figure S1. Calibration plot of areas (mean) *versus* rivastigmine concentration ($n = 3$).

The R^2 obtained was almost 1, which means that the linearity obtained is adequate for rivastigmine quantification within the evaluated concentration range.

2.3. Precision

Repeatability or intra-day precision and intermediate precision or inter-day precision were analysed for three different concentrations of rivastigmine injected in three different days. The results are showed in Table S2. Instrumental precision was also analysed for standard and sample and results are presented in Table S3.

Table S2. Results achieved for the intra-day precision and inter-day precision.

	Intra-day[a]				Inter-day[b]		
Day	Rivastigmine[c] (µg/mL)	Rivastigmine[d] (µg/mL)	SD	RDS (%)	Rivastigmine[d] (µg/mL)	SD	RDS (%)
1	48	44.7077	0.0026	0.3105			
2	48	44.9897	0.0006	0.0675	44.9897	0.0006	0.0674
3	48	44.7333	0.0020	0.1792			
1	120	119.5023	0.0011	0.0633			
2	120	119.2718	0.0038	0.2079	119.2718	0.0040	0.2079
3	120	118.7077	0.0131	0.7229			
1	1200	1149.9897	0.0114	0.0746			
2	1200	1152.9128	0.0080	0.0526	1152.9128	0.0080	0.0526
3	1200	1164.6564	0.0035	0.0228			

[a] $n = 3$; [b] $n = 9$; [c] Rivastigmine (µg/mL); [d] Mean values of rivastigmine concentration.

Table S3. Results obtained for the instrumental precision.

Rivastigmine[a] (µg/mL)		
	**Standard**	**Rivastigmine-loaded NLC supernatant**
	1164.6308	21.6061
	1164.9385	21.6985
	1164.0923	21.7292
	1164.4000	22.0677
	1167.4769	21.9446
	1165.8615	21.9754
	1167.7077	21.7600
	1163.8615	21.8215
	1162.4769	22.0984
	1162.7846	22.1292
**Mean**	1164.8231	21.8831
**SD**	1.7572	0.1846
**RSD (%)**	0.1509	0.8436

[a] Mean measured rivastigmine concentration.

The results presented in Table S2 showed the existence of intra-day and inter-day precision, as indicated by the RSD values under 1.0%. The instrumental precision was also satisfactory for rivastigmine-loaded NLC supernatant, which also showed RDS below 1%.

2.4. Specificity

The specificity was evaluated comparing the chromatogram of supernatant of a placebo NLC formulation (Figure S2) with the chromatogram of a 1200 µg/mL rivastigmine standard solution (Figure S3). As can be observed from Figures S2 and S3, the supernatant of placebo NLC formulation did not exhibited any peak at the drug retention time. Thus, the method was considered specific.

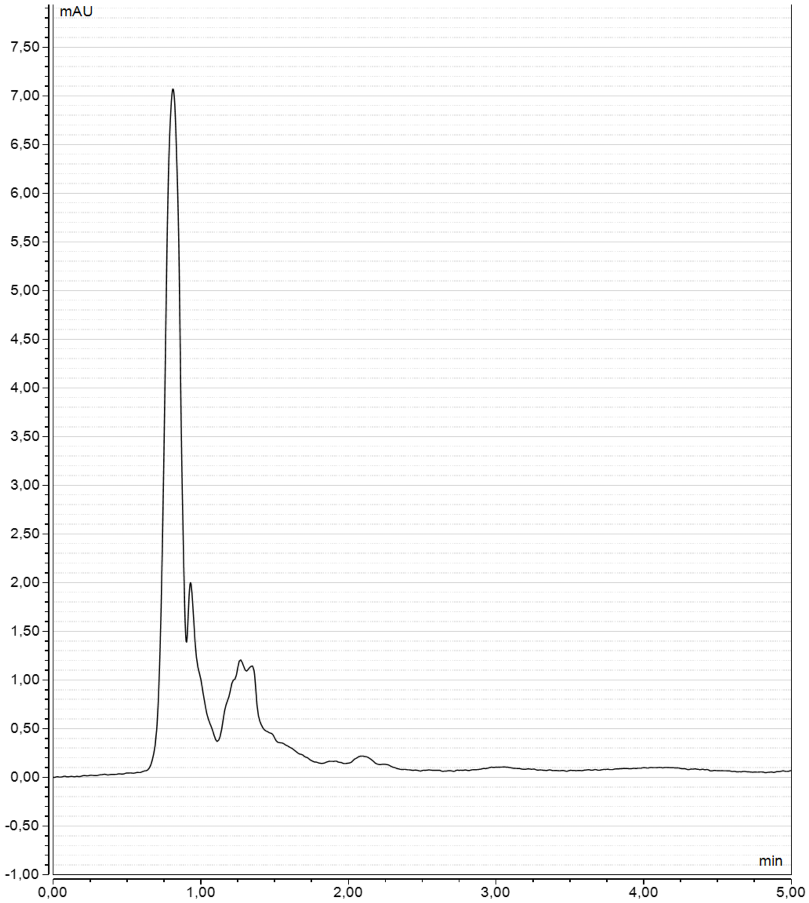

Figure S2. Chromatogram of the supernatant of placebo-NLC formulation.

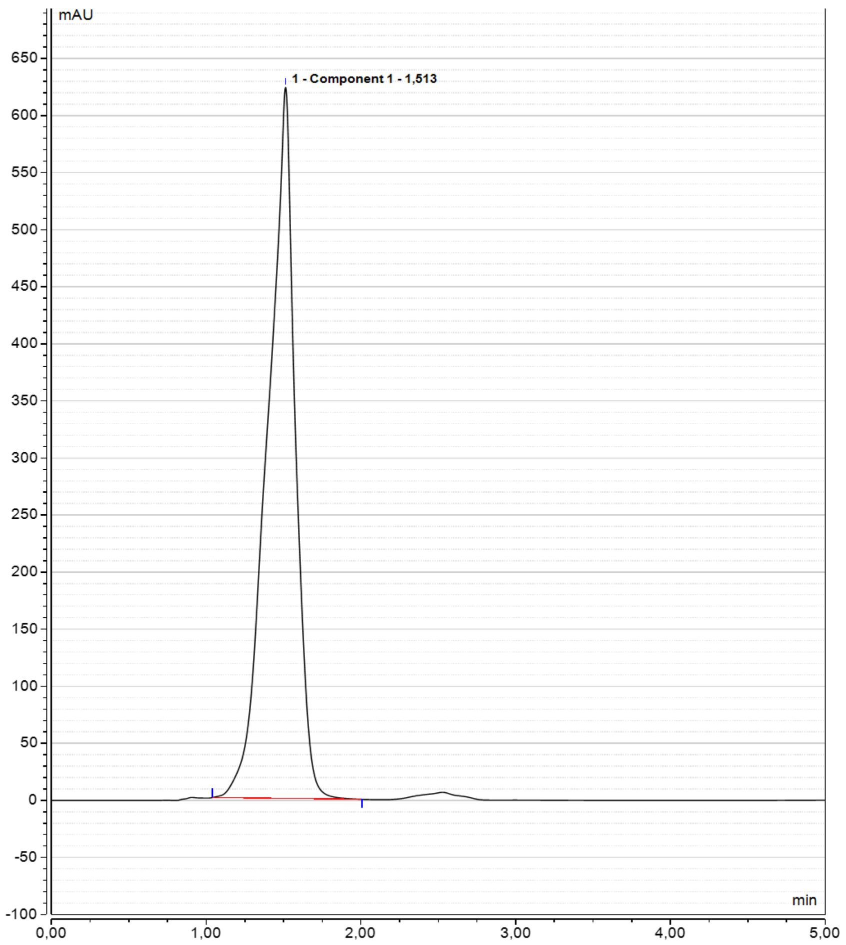

Figure S3. Chromatogram of standard 1200 µg/mL rivastigmine solution.

2.5. Accuracy

The percentage of drug recovery was calculated for three intermediate points of the calibration curve: low, intermediate, and high (48, 120 and 1200 µg/ mL, respectively) (Table S4).

Table S4. Drug recovery for method accuracy.

Rivastigmine (µg/mL)	Recovery[a] (%)	RSD[b] (%)
**48**	103.0009	0.2176
**120**	96.7987	0.5342
**1200**	99.1819	0.2911

[a] Mean measured/added rivastigmine concentration ×100; [b] *n* = 3.

The values of recovery for the three standard solutions were compared with accuracy results reported from the same drug in cerebrospinal fluid and blood serum and for other acetylcholinesterase inhibitor drug (donepezil) in plasma, due to the lack of literature related to a validated HPLC method for pure rivastigmine [7, 8]. The mean recovery values were close to 100 % and the RSD was less than 2 %, indicating low variability and strong agreement between the experimental and theoretical concentration values.

2.6. Detection Limit (DL) and Quantification Limit (QL)

The DL and QL of rivastigmine were calculated from the standard calibration curve and standard deviation of the response. The results are shown in Table S5.

Table S5. Detection and quantification limits.

Detection and quantification limits	
**DL**	**0.5619** μg/mL
**QL**	1.7028 ml

2.7. Robustness

Table S6 shows the results of the effect of three different flow rates in the retention time, recovery and RDS.

Table S6. Results of the method robustness after variation the flow rate.

		Retention time (min)	Recover [a] (%)	RDS [b] (%)
	0.5	2.6170	99.5153	0.1396
**Flow rate**	1.5	1.0470	101.0590	0.0250
	1.75	0.7870	94.0288	0.7448

[a] Mean value of rivastigmine concentration ×100; [b] $n = 3$.

Table S7 shows the results of the variations on the concentration of the mobile phase.

Table S7. Results of the method robustness after variations in the mobile phase.

		Retention time (min)	Recover [a] (%)	RDS [b] (%)
**Mobile phase (acetonitrile/buffer phosphate, %)**	50:50	1.3400	97.8993	0.0160
	40:60	1.2400	99.2231	0.9471
	70:30	1.5470	101.9896	0.3009

[a] Mean value of rivastigmine concentration × 100; [b] n=3.

The values of recovery and RSD showed that no significant alterations were observed after small variations, being the method considered robust.

2.8. Method Applicability

Assessment of encapsulation efficiency (EE) and loading capacity (LC) parameters of rivastigmine-loaded NLC formulations.

Table S8. Results of encapsulation efficiency (EE) and loading capacity (LC) of rivastigmine-loaded NLC formulations.

Formulation	Encapsulation efficiency (EE) (%)	Loading Capacity (LC) (%)
1	92.122	10.174
2	92.122	10.174
3	92.030	10.165
4	91.994	10.161
5	91.881	10.150
6	91.732	10.135
7	91.573	10.119
8	91.522	10.114
9	91.517	10.114
10	91.414	10.104
**Mean**	91.791	10.141
**SD**	0.272	0.027
**RSD (%)**	0.296	0.266

The values of EE and LC revealed that the NLC are effective for rivastigmine encapsulation. Furthermore, the high EE confirmed the solubility of rivastigmine in the lipids used for the preparation of NLC [9].

3. Design of Experiment (DoE) for Rivastigmine-Loaded NLC

3.1. Part 1: Optimization of Formulation Variables by Central Composite Design (CCD)

Table S9. ANOVA models and respective R squared (R^2).

	R^2					
**ANOVA models**	**Z-Ave**[1]	**D50**[2]	**D90**[2]	**PDI**[3]	ZP[4]	EE[5]
Linear main effects only	0.528	0.522	0.510	0.159	0.492	0.176
Lin./quad. main effects	0.813	0.699	0.773	0.657	0.854	0.736
Linear main effects. + 2-ways	0.529	0.749	0.674	0.227	0.570	0.177
Lin./quad main eff. + 2-ways	0.815	0.926	0.936	0.725	0.932	0.737

[1]Z-Ave (mean particle size, nm); [2]volume distribution (D50 and D90, nm); [3]PDI (polydispersity index); [4]ZP (zeta potential, mV) and [5]EE (encapsulation efficiency, %).

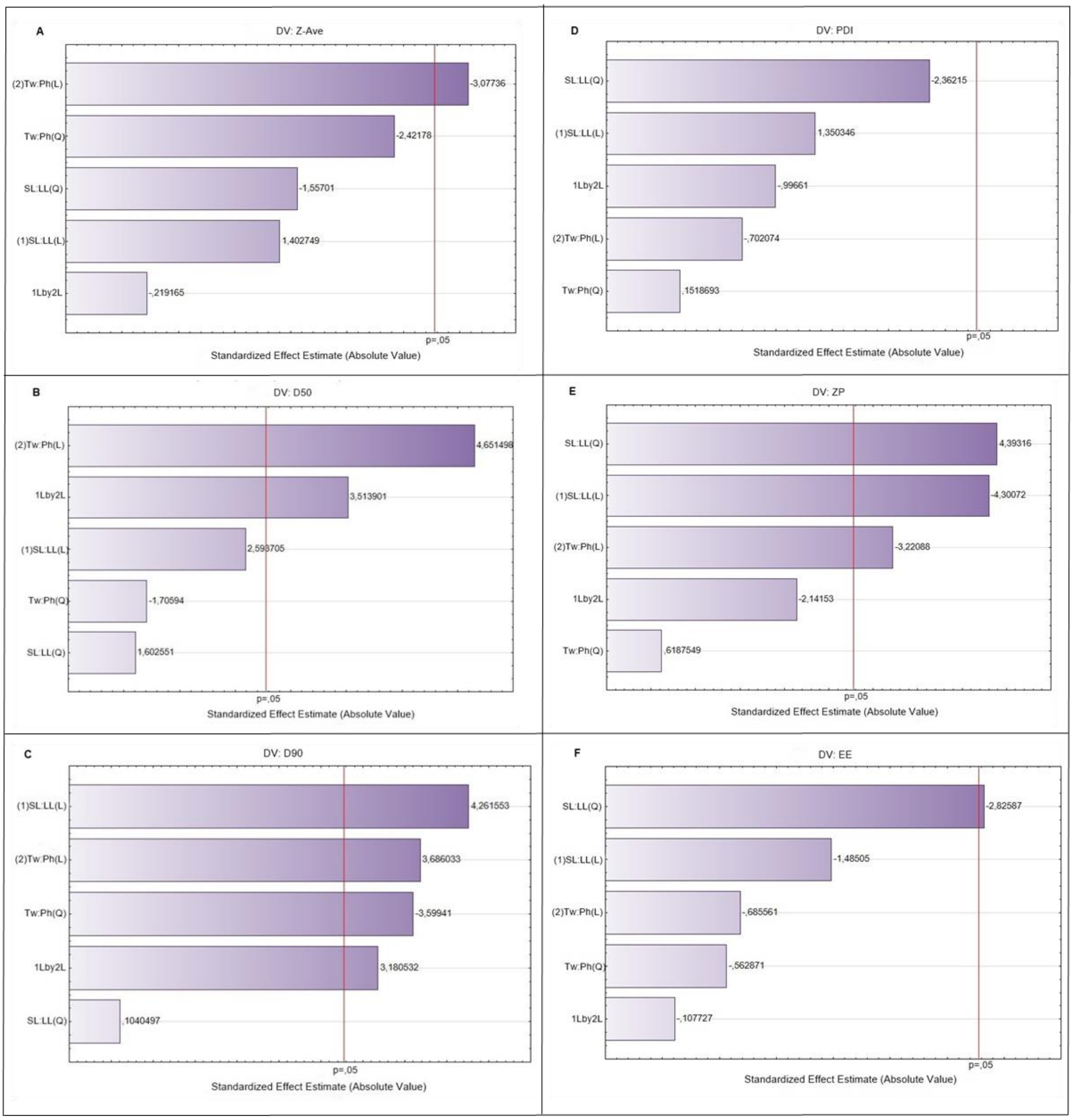

Figure S4. Pareto chart showing the effects of CMAs on CQAs, *viz.*, size (Z-Ave, D50 and D90) (left: A-C), PDI, ZP and EE (right: D-F).

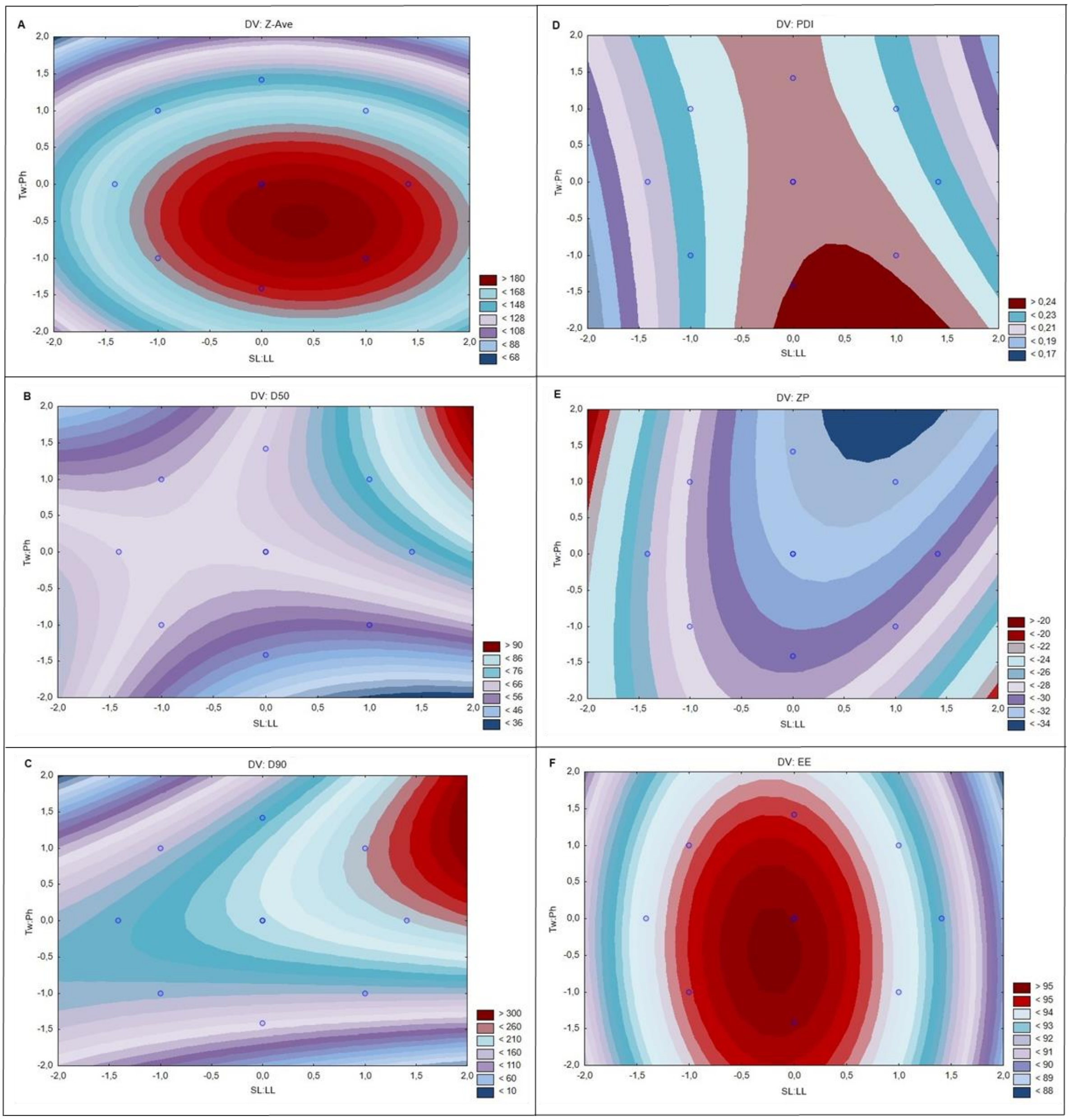

Figure S5. Contour plot for CQAs, *viz.*, size (Z-Ave, D50 and D90) (left: A-C); and PDI, ZP and EE (right: D-F).

Screening of drug and excipients

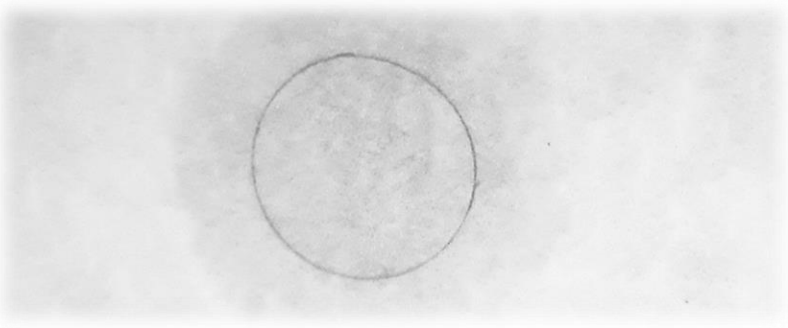

Figure S6. Filter paper showing the results of screening of drugs and lipids, where the absence of oil droplets resulting from the solubilisation of drug in the lipid mixture is observed.

3.2. Part 2: optimization of instrumental parameters by Box-Behnken design (BBD)

3.2.1. Effect of emulsification speed and HPH cycles on size (Z-Ave, D50 and D90), PDI, ZP and EE

Table S10. ANOVA models and respective R^2 for instrumental parameters: emulsification speed and number of HPH cycles.

	R^2					
**ANOVA models**	**Z-Ave**[1]	**D50**[2]	**D90**[2]	**PDI**[3]	ZP[4]	EE[5]
No interactions	0.973	0.662	0.980	0.609	0.795	0.303
2-ways interactions (linear × linear)	0.976	0.830	0.980	0.621	0.873	0.439
2-ways interactions (linear. quadr.)	1.000	1.000	1.000	1.000	1.000	1.000

[1]Z-Ave (mean particle size, nm); [2]volume distribution (D50 and D90, nm); [3]PDI (polydispersity index); [4]ZP (zeta potential, mV) and [5]EE (encapsulation efficiency, %).

3.2.2. Effect of ultrasound technique on size (Z-Ave, D50 and D90), PDI, ZP and EE

Table S11. ANOVA models and respective R^2 for instrumental parameter: sonication amplitude.

	R^2					
**ANOVA models**	**Z-Ave**[1]	**D50**[2]	**D90**[2]	**PDI**[3]	ZP[4]	EE[5]
No interactions	0.888	0.954	0.940	0.898	0.833	0.826
2-ways interactions (linear × linear)	0.972	0.964	0.965	0.906	0.837	0.857
2-ways interactions (linear. quadr.)	1.000	1.000	1.000	1.000	1.000	1.000

[1]Z-Ave (mean particle size, nm); [2]volume distribution (D50 and D90, nm); [3]PDI (polydispersity index); [4]ZP (zeta potential, mV) and [5]EE (encapsulation efficiency, %).

4. pH and Osmolarity

Table S12. Critical quality attributes (CQAs) values of rivastigmine-loaded NLC formulations before and after the pH and osmolarity adjustment by addition of HCl and glycerin.

**Before addition**	**Ultrasound technique**	**HPH method**
Z-Ave[1] (nm)	112.610±0.613	111.670±0.710
D50[2] (nm)	69.712±0.710	52.242±0.244
D90[2] (nm)	123.011±1.712	119.121±0.642
PDI[3]	0.219±0.002	0.196±0.007
ZP[4] (mV)	-29.921±0.433	-30.120±0.610
EE[5] (%)	97.536±0.249	98.122±0.432
**After addition**		
Z-Ave[1] (nm)	114.000±1.910	109.000±0.850
D50[2] (nm)	70.126±0.341	60.220±0.392
D90[2] (nm)	130.440±1.120	126.100±1.010
PDI[3]	0.221±0.003	0.196±0.007
ZP[4](mV)	-30.633±0.288	-30.466±0.252
EE[5](%)	96.987±0.446	97.174±0.297

[1]Z-Ave (mean particle size, nm); [2]volume distribution (D50 and D90, nm); [3]PDI (polydispersity index); [4]ZP (zeta potential, mV) and [5]EE (encapsulation efficiency, %). Results presented as mean ± SD (n = 3).

References

1. Fazil, M., et al., *Development and evaluation of rivastigmine loaded chitosan nanoparticles for brain targeting.* European journal of pharmaceutical sciences, 2012. **47**(1): p. 6-15.
2. Guideline, I.H.T. *Validation of analytical procedures: text and methodology Q2 (R1).* in *International conference on harmonization, Geneva, Switzerland*. 2005.

3. Commission, E.P., E.D.f.t.Q.o. Medicines, and Healthcare, *European pharmacopoeia*. Vol. 1. 2010: Council of Europe.
4. Dolan, J.W., *Why do peaks tail.* LCGC Eur, 2003. **21**(7): p. 610-613.
5. Rao, B.M., et al., *A stability indicating LC method for rivastigmine hydrogen tartrate.* Journal of pharmaceutical and biomedical analysis, 2005. **37**(1): p. 57-63.
6. Cacicedo, M., et al., *Lipid nanoparticles–Metvan: reveling a novel way to deliver a vanadium compound to bone cancer cells.* New Journal of Chemistry, 2019.
7. Petrocheilou, M., et al., *A simple and direct HPLC-DAD method for the simultaneous determination of galantamine, donepezil and rivastigmine in cerebrospinal fluid, blood serum and urine.* Journal of Applied Bioanalysis, 2017. **3**(4): p. 1847.
8. Hanafy, D.M., et al., *Biophenols of mints: Antioxidant, acetylcholinesterase, butyrylcholinesterase and histone deacetylase inhibition activities targeting Alzheimer's disease treatment.* Journal of functional foods, 2017. **33**: p. 345-362.
9. Shah, B., et al., *Intranasal delivery of venlafaxine loaded nanostructured lipid carrier: Risk assessment and QbD based optimization.* Journal of Drug Delivery Science and Technology, 2016. **33**: p. 37-50.

© 2020 by the authors. Submitted for possible open access publication under the terms and conditions of the Creative Commons Attribution (CC BY) license (http://creativecommons.org/licenses/by/4.0/).
